# HTLV-1 induces an inflammatory CD4^+^CD8^+^ T cell population in HTLV-1–associated myelopathy

**DOI:** 10.1172/jci.insight.173738

**Published:** 2024-01-09

**Authors:** Allison K. Maher, Aris Aristodemou, Nicolas Giang, Yuetsu Tanaka, Charles R.M. Bangham, Graham P. Taylor, Margarita Dominguez-Villar

**Affiliations:** 1 Department of Infectious Disease, Faculty of Medicine, Imperial College London, London, United Kingdom.; 2 Laboratory of Hematoimmunology, Graduate School of Health Sciences, University of the Ryukyus, Nishihara, Okinawa, Japan.

**Keywords:** Immunology, Adaptive immunity, T cells

## Abstract

Human T cell leukemia virus type 1 (HTLV-1) is a retrovirus with preferential CD4^+^ T cell tropism that causes a range of conditions spanning from asymptomatic infection to adult T cell leukemia and HTLV-1–associated myelopathy (HAM), an inflammatory disease of the CNS. The mechanisms by which HTLV-1 induces HAM are poorly understood. By directly examining the ex vivo phenotype and function of T cells from asymptomatic carriers and patients with HAM, we show that patients with HAM have a higher frequency of CD4^+^CD8^+^ double-positive (DP) T cells, which are infected with HTLV-1 at higher rates than CD4^+^ T cells. Displaying both helper and cytotoxic phenotypes, these DP T cells are highly proinflammatory and contain high frequencies of HTLV-1–specific cells. Mechanistically, we demonstrate that DP T cells arise by direct HTLV-1 infection of CD4^+^ and CD8^+^ T cells. High levels of CD49d and CXCR3 expression suggest that DP T cells possess the ability to migrate to the CNS, and when cocultured with astrocytes, DP T cells induce proinflammatory astrocytes that express high levels of CXCL10, IFN-γ, and IL-6. These results demonstrate the potential of DP T cells to directly contribute to CNS pathology.

## Abstract 2

Patients with HTLV-1-associated myelopathy display an increased frequency of CD4^+^CD8^+^ double positive T cells with an inflammatory phenotype driven by HTLV-1 infection of single positive T cells.

## Graphical abstract

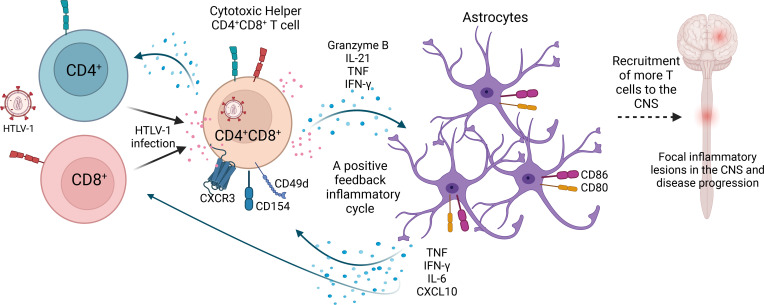

## Introduction

Human T cell leukemia virus type 1 (HTLV-1) is a retrovirus that infects at least 5 million to 10 million people worldwide (1–3). Although the majority of HTLV-1–infected individuals remain asymptomatic carriers (ACs), infection results in a 57% increase in adjusted mortality rate. A range of 2%–5% of HTLV-1 carriers develop adult T cell leukemia, and another 2%–5% develop inflammatory diseases, including HTLV-1–associated myelopathy (HAM) (1, 4). HAM is an inflammatory disease of the CNS characterized by chronic inflammatory demyelination that resembles progressive spinal forms of multiple sclerosis (MS) (5, 6). T cells are the main target of HTLV-1 infection, and CD4^+^ and CD8^+^ T cells are thought to respectively carry 95% and 5% of the HTLV-1 proviral load (PVL) in an infected individual (7). There are currently no readily available cures for any of the HTLV-1–associated diseases, and HAM treatment aims to reduce the severity of the symptoms by reducing inflammation; however, its efficacy is limited (1). Individuals with higher HTLV-1 PVLs are more likely to develop HAM (8–10), although it is unclear why all HTLV-1 carriers with high PVL do not progress to HAM. While the exact mechanisms of HAM pathogenesis are unknown, the current understanding is that an inefficient immune response to HTLV-1 results in a high PVL and chronic high levels of HTLV-1 antigen expression (1, 11, 12). This results in chronically activated CD4^+^ T cells that invade the CNS and persistently secrete proinflammatory cytokines, which are neurotoxic at high concentration (13). A positive feedback loop recruits more CD4^+^ and CD8^+^ T cells to the CNS, eventually causing focal inflammatory lesions in the CNS (14, 15).

Mature double-positive (DP) T cells, expressing both CD4 and CD8, account for approximately 1%–3% of peripheral T cells in healthy individuals (16–18). Increased frequencies of DP T cells have been reported in autoimmune diseases such as MS, myasthenia gravis, and rheumatoid arthritis (RA) as well as in viral infections such as HIV, Hepatitis C virus (HCV), and Epstein-Barr virus (EBV) (19–24). DP T cells are phenotypically heterogeneous and have been scarcely studied, with contrasting results on their effector, regulatory, and cytotoxic functions in different diseases (25–30).

Here we show that patients with HAM have a higher frequency of peripheral DP T cells than asymptomatic carriers with a similar PVL. Moreover, DP T cells are infected with HTLV-1 at a higher rate than CD4^+^ or CD8^+^ single-positive (SP) T cells and account for approximately 8% of the total HTLV-1 PVL. In HTLV-1–infected individuals, DP T cells are proinflammatory and express high levels of IFN-γ, TNF, IL-17A, IL-21, and granzyme B, suggesting both cytotoxic and helper phenotypes, and they contain increased frequencies of HTLV-1–specific cells as compared with their CD4^+^ and CD8^+^ SP counterparts. Mechanistically, we demonstrate that HTLV-1 infection of SP T cells triggers the emergence of DP T cell populations from both CD4^+^ and CD8^+^ SP T cells in vitro by modulating the expression of ThPOK and RunX3 transcription factors. We show that chemokine and integrin receptors involved in lymphocyte migration to the CNS, including CD49d and CXCR3, are more highly expressed by DP T cells than by CD4^+^ and CD8^+^ SP cells, suggesting that DP T cells possess an enhanced ability to migrate to the CNS. Finally, DP T cells induce proinflammatory astrocytes in vitro that express high levels of CXCL10, IFN-γ, and IL-6, indicating that DP T cells have the capacity to directly contribute to CNS pathology.

## Results

### Patients with HAM have an increased frequency of DP T cells.

**Figure 1:**
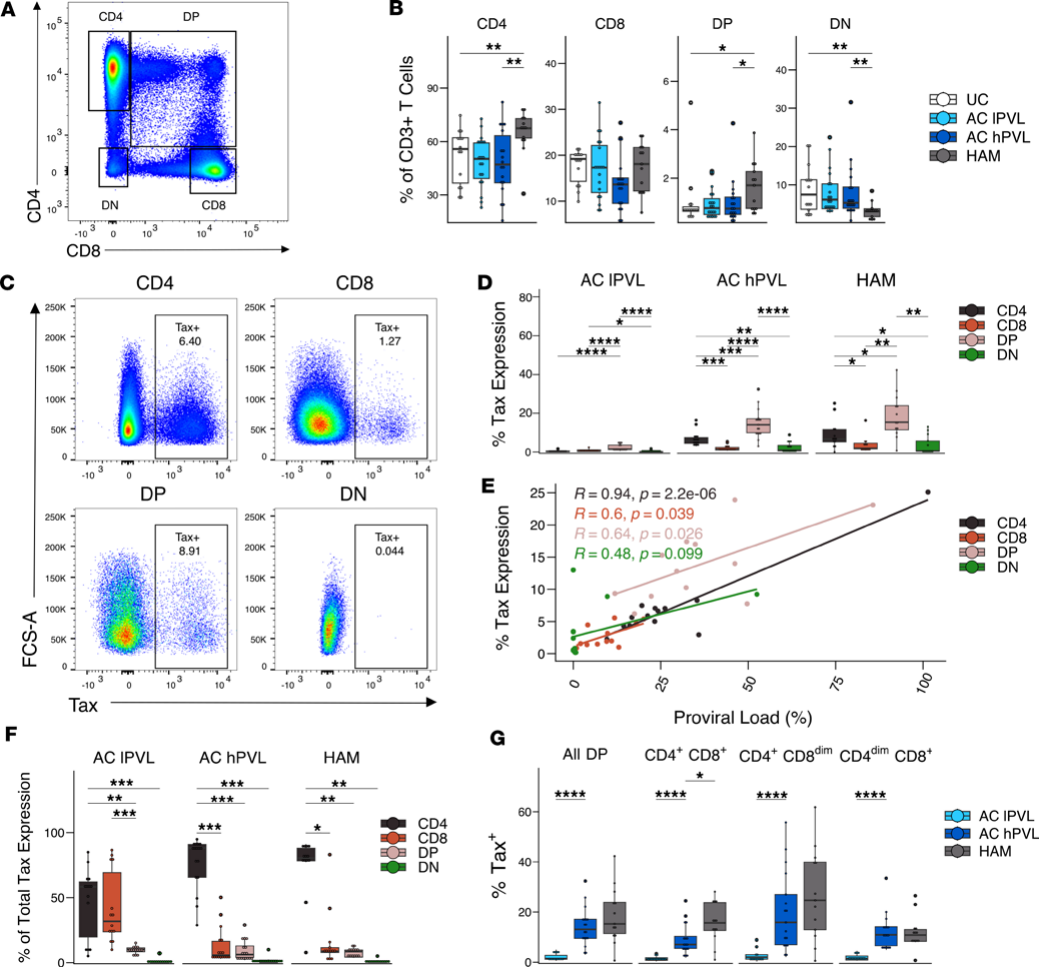
Frequency and HTLV-1 infection rate of DP T cells. (**A**) Representative flow plot of CD4 and CD8 expression in viable singlet CD3^+^ cells in a HTLV-1 carrier showing CD4^+^, CD8^+^, double-negative (DN), and double-positive (DP) T cells. (**B**) Box-and-whisker plot displaying the frequencies of peripheral T cell populations (*n* = 14 UC, *n* = 19 AC lPVL, *n* = 18 AC hPVL, *n* = 13 HAM). (**C** and **D**) Representative flow plot (**C**) and box-and-whisker plot (**D**) showing Tax expression in different T cell populations after 12 hours in culture (*n* = 15 AC lPVL, *n* = 17 AC hPVL, *n* = 13 HAM). (**E**) Scatter plot of Tax expression after 12 hours in culture versus the proviral load measured by ddPCR (CD4 and DN: *n* = 7 AC hPVL, *n* = 6 HAM; CD8 and DP: *n* = 7 AC hPVL, *n* = 5 HAM). (**F**) Box-and-whisker plot displaying the contribution of CD4^+^ SP, CD8^+^ SP, DP, and DN T cells to total Tax expression in CD3^+^ T cells (*n* = 15 AC lPVL, *n* = 16 AC hPVL, *n* = 13 HAM). (**G**) Box-and-whisker plot of Tax expression in DP T cell subpopulations (*n* = 15 AC lPVL, *n* = 16 AC hPVL, *n* = 13 HAM). Wilcoxon signed-rank unpaired test for comparisons between UC and AC lPVL, UC and HAM, AC lPVL and AC hPVL, and AC hPVL and HAM (**B**); Wilcoxon signed-rank paired test with Holm correction for all possible multiple comparisons (**D**); Pearson correlation coefficient (R) and *P* value (**E**); Wilcoxon signed-rank paired test with Holm correction for comparisons between CD4 and CD8, CD4 and DP, and CD8 and DP (**F**); Wilcoxon signed-rank unpaired test for comparisons between AC lPVL and AC hPVL, and AC hPVL and HAM (**G**). *P < 0.05, **P < 0.01, ***P < 0.001, ****P < 0.0001.

We initially examined the ex vivo frequencies of peripheral CD4^+^ SP, CD8^+^ SP, DP, and double-negative (DN) T cells in HTLV-1–infected individuals and healthy uninfected controls (UCs) (Figure 1A). HTLV-1–infected individuals were grouped into ACs with a PVL under 3% (low PVL [AC lPVL]), ACs with a PVL above 3% (high PVL [AC hPVL]), and patients with HAM. The PVLs of the ACs with a high PVL and patients with HAM were matched to control for potential differences due to HTLV-1 infection (Supplemental Figure 1A; supplemental material available online with this article; https://doi.org/10.1172/jci.insight.173738DS1). Patients with HAM displayed a higher frequency of DP T cells compared with AC and UCs (Figure 1B). CD3^+^ T cells from patients with HAM also contained higher frequencies of CD4^+^ SP T cells and a lower frequency of DN T cells than both ACs and UCs. DP T cells displayed varying levels of CD4^+^ and CD8^+^ expression. Thus, 3 subpopulations could be identified — i.e., CD4^+^CD8^dim^, CD8^+^CD4^dim^, and CD4^+^CD8^+^ (CD4^bright^CD8^bright^) (Supplemental Figure 1B), with CD4^+^CD8^dim^ T cells being present in the highest frequency in all patient groups (Supplemental Figure 1C). The higher frequency of DP T cells in patients with HAM was mainly driven by increased frequencies of CD4^+^CD8^dim^ and CD4^dim^CD8^+^ T cells.

To determine whether DP T cells were infected by HTLV-1, we examined the expression of the HTLV-1 protein Tax after 12 hours in culture (Figure 1C). The frequency of Tax-expressing cells was higher in DP T cells as compared with CD4^+^ SP T cells (which are the main HTLV-1 reservoir; ref. 7), CD8^+^ SP T cells, and DN T cells in all HTLV-1–infected groups (Figure 1D). In AC hPVL and HAM groups, there was a 2-fold higher frequency of Tax expression in DP cells as compared with CD4^+^ SP T cells (6.65% and 8.84% Tax^+^ cells in CD4^+^ SP T cells in AC hPVL and HAM, respectively, compared with 14.1% and 18.3% Tax^+^ cells in DP T cells, respectively). Although Tax is expressed dynamically by infected cells (1, 31), in our experimental setting, Tax expression in T cells after 12 hours in culture strongly correlated with the PVL of sorted CD4^+^ SP, CD8^+^ SP, DP, and DN T cells measured by digital droplet PCR (ddPCR; Figure 1E) (32); this allowed us to use Tax expression at 12 hours as a reliable readout for PVL and infection rate. Given that Tax expression correlated with PVL in all T cell populations, the high levels of Tax expression in DP T cells were not due to different Tax expression dynamics in DP T cells (Figure 1E). The mean DP T cell contribution in all patient groups to the total proviral burden was 8.2%, despite DP T cells accounting for less than 2% of the total CD3^+^ T cell population (Figure 1F). In AC hPVL and patients with HAM, CD8^+^ SP T cells and DP T cells contributed equally to the total HTLV-1 proviral burden. While the majority of the HTLV-1 PVL in high proviral groups was carried in CD4^+^ SP T cells as expected (7), the proviral burden in AC lPVL individuals was distributed more evenly between CD4^+^ and CD8^+^ SP T cells with 47% and 42% of the total Tax expression, respectively. HAM and AC hPVL did not differ in their PVL distribution, and CD4^+^ and CD8^+^ SP T cells expressed, on average, 78% and 13% of the total Tax, respectively (Supplemental Figure 1D). Moreover, high rates of Tax expression were seen in all DP T cell subpopulations (Figure 1G). Patients with HAM displayed significantly higher rates of HTLV-1 infection in CD4^+^CD8^+^ T cells than AC hPVLs, and there was a similar trend in CD4^+^CD8^dim^ T cells, which harbored the highest rate of infection of all DP T cell subpopulations. Overall, our data indicate that DP T cells are more abundant in patients with HAM compared with ACs and are infected with HTLV-1 at higher rates than all other T cell populations.

### DP T cells are a distinct T cell population in asymptomatic HTLV-1 carriers.

**Figure 2:**
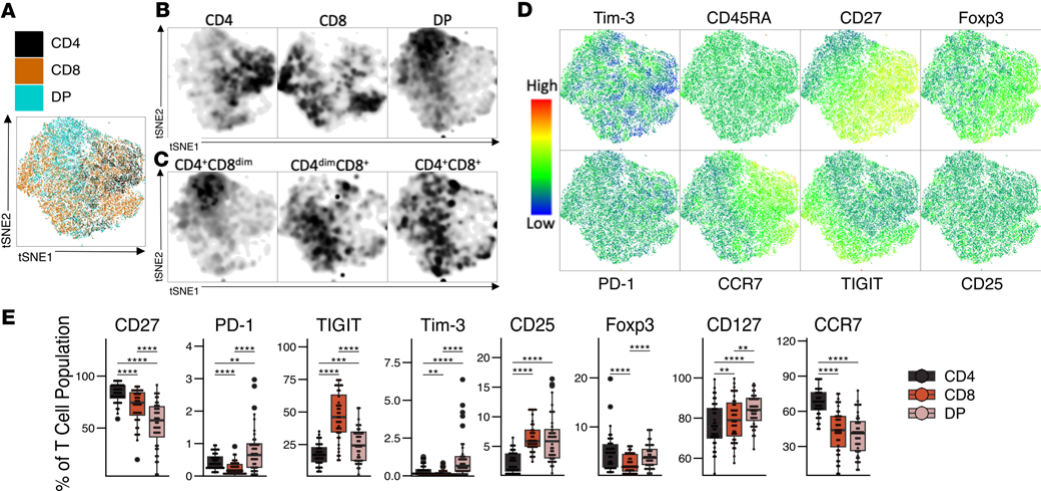
Characterization of DP T cells in HTLV-1 asymptomatic carriers. (**A**) t-SNE plot obtained from a concatenated sample of ex vivo CD3^+^ T cells (*n* = 14 AC lPVL, *n* = 14 AC hPVL). (**B** and **C**) t-SNE plot showing the density of CD4^+^ SP, CD8^+^ SP, and DP (**B**) or CD4^+^CD8^dim^, CD4^dim^CD8^+^, and CD4^+^CD8^+^ T cells (**C**). (**D**) t-SNE plots displaying the levels of expression of various markers. (**E**) Box-and-whisker plot showing the percentage of expression of markers in ex vivo CD4^+^ SP, CD8^+^ SP, and DP T cells from asymptomatic carriers (*n* = 20 AC lPVL, *n* = 20 AC hPVL). (**E**) Wilcoxon signed-rank paired test with Holm correction for multiple comparisons. **P < 0.01, ***P < 0.001, ****P < 0.0001.

Given the high rate of HTLV-1 infection of DP T cells, we characterized the phenotype of DP T cells in AC by high dimensional flow cytometry and compared them with CD4^+^ and CD8^+^ SP T cells. We examined the expression of Tim-3, PD-1, TIGIT, CD25, Foxp3, CCR7, CD45RA, and CD27 as markers of exhaustion, activation, and antigen experience. Dimensionality reduction demonstrated that the phenotype of DP T cells was different from those of CD4^+^ and CD8^+^ SP T cells on a t-distributed stochastic neighbor embedding (t-SNE) plot (Figure 2, A and B). In addition, the DP T cell subpopulations also partially clustered separately from each other (Figure 2C). The main markers driving the clustering were CD27, Foxp3, PD-1, TIGIT, and CCR7 (Figure 2D). Relative to both SP T cell populations, DP T cells expressed less CD27 and more PD-1, Tim-3, and CD127 (Figure 2E and Supplemental Figure 2). DP T cells expressed more TIGIT and CD25 than CD4^+^ SP T cells, but the level of these markers was lower in the case of TIGIT or equal to, in the case of CD25, than on CD8^+^ SP T cells. In addition, DP T cells expressed similar levels of Foxp3 as did CD4^+^ SP T cells and higher levels than CD8^+^ SP T cells, but they expressed lower levers of the lymph node–homing marker CCR7 than CD4^+^ SP T cells.

**Figure 3:**
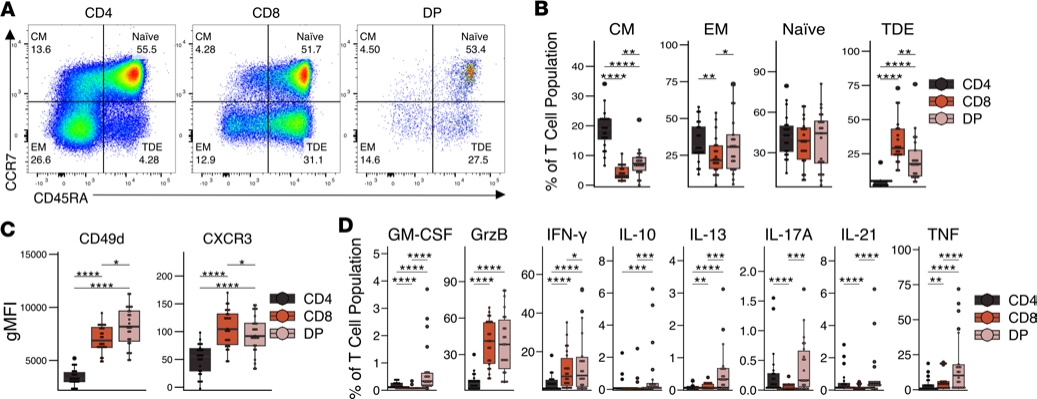
Characterization of DP T cell antigen experience and cytokine expression in HTLV-1 asymptomatic carriers. (**A** and **B**) A representative flow plot showing (**A**) and box-and-whisker plot (**B**) showing the frequency of central memory (CM), naive, terminally differentiated effector (TDE), and effector memory (EM) in DP T cells (*n* = 10 AC lPVL, *n* = 11 AC hPVL). (**C**) Box-and-whisker plot showing the geometric mean fluorescence intensity (gMFI) of CD49d and CXCR3 (*n* = 12 AC lPVL, *n* = 14 AC hPVL). (**D**) Box-and-whisker plot of the expression of cytokines and granzyme B after a 4 hour stimulation with PMA and ionomycin (*n* = 11 AC lPVL, *n* = 11 AC hPVL). Wilcoxon signed-rank paired test with Holm correction for multiple comparisons (**B** and **D**); *t* test with Holm correction for multiple comparisons (**C**). *P < 0.05, **P < 0.01, ***P < 0.001, ****P < 0.0001.

Approximately 50% of the DP T cells had a naive (CCR7^+^CD45RA^+^) phenotype (Figure 3A), similar to their respective CD4^+^ and CD8^+^ SP T cell populations (Figure 3B). In contrast, DP cells displayed a distinct distribution of memory phenotypes compared with SP CD4^+^ and CD8^+^ T cells, and while DP cells contained a higher frequency of CM (CCR7^+^CD45RA^–^) and EM (CCR7^–^CD45RA^–^) than CD8^+^ SP T cells, this frequency was lower than in CD4^+^ SP T cells. A larger proportion of DP T cells had a terminally differentiated effector (TDE) phenotype (CCR7^–^CD45RA^+^) compared with CD4^+^ SP T cells, but that proportion was smaller than in CD8^+^ SP T cells. While DP T cells expressed lower levels of the lymph node–homing marker CCR7 than CD4^+^ SP T cells (Figure 2E), they expressed higher levels of the tissue-homing marker CD49d than both SP T cell populations and higher levels of CXCR3 than CD4^+^ SP T cells (Figure 3C), suggesting an increased migratory capacity to tissues.

To explore the functionality of DP T cells in ACs, we determined the cytokine expression profile ex vivo after a 4-hour stimulation with PMA and ionomycin (Figure 3D and Supplemental Figure 3). DP T cells displayed a proinflammatory phenotype clearly different from their SP CD4^+^ and CD8^+^ T cell counterparts. DP T cells expressed high levels of TNF and comparable levels of IFN-γ and granzyme B to those of CD8^+^ SP T cells; they expressed levels of IL-17A and IL-21 similar to those of CD4^+^ SP T cells. DP T cells also expressed higher levels of GM-CSF, IL-10, and IL-13 than either SP T cell population, but the overall expression was low. These data suggest that DP T cells are polyfunctional and proinflammatory and that they display both helper and cytotoxic phenotypes in HTLV-1 ACs.

### Contribution of DP T cells to anti–HTLV-1 immune response.

**Figure 4:**
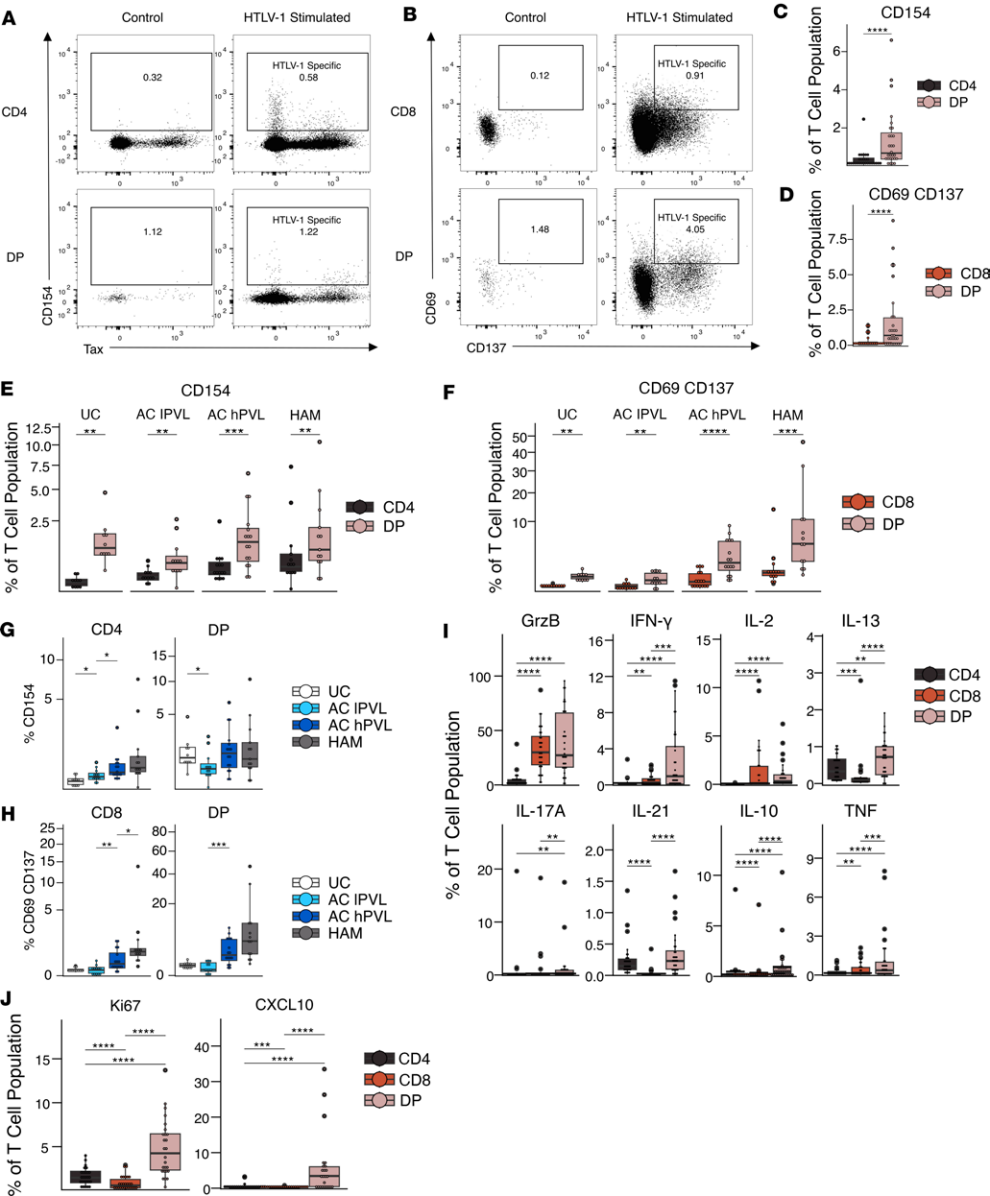
Contribution of DP T cells to HTLV-1–specific immunity. (**A** and **B**) Representative flow plots showing the expression of CD154 and Tax by CD4^+^ SP and DP T cells (**A**) and the expression of CD69 and CD137 by CD8^+^ SP T and DP T cells (**B**) from a high proviral load asymptomatic carrier after a 12-hour stimulation with HTLV-1. (**C** and **D**) Box-and-whisker plot showing the frequency of CD4^+^ SP and DP T cells expressing CD154 (**C**) and CD8^+^ SP and DP T cells coexpressing CD69 and CD137 (**D**) in asymptomatic carriers (*n* = 12 AC lPVL, *n* = 16 AC hPVL). (**E** and **F**) Box-and-whisker plot showing the frequency of CD4^+^ SP and DP T cells expressing CD154 (**E**) or CD8^+^ SP and DP T cells coexpressing CD69 and CD137 (**F**) in different patient groups (*n* = 10 UC, *n* = 12 AC lPVL, *n* = 16 AC hPVL, *n* = 13 HAM). (**G** and **H**) Box-and-whisker plot comparing the frequency of CD154^+^ CD4^+^ SP and DP T cells (**G**) or CD69^+^CD137^+^ CD8^+^ SP and DP T cells (**H**) among patient groups (*n* = 10 UC, *n* = 12 AC lPVL, *n* = 16 AC hPVL, *n* = 13 HAM). (**I**) Box-and-whisker plot of the expression of cytokines and granzyme B in CD4^+^ SP, CD8^+^ SP, and DP T cells from asymptomatic carriers after stimulation with heat-inactivated HTLV-1 for 20 hours (*n* = 17 AC lPVL, *n* = 16 AC hPVL). (**J**) Box-and-whisker plot showing Ki67 and CXCL10 expression in CD4^+^ SP, CD8^+^ SP, and DP T cells from asymptomatic carriers after stimulation with heat-inactivated HTLV-1 for 12 hours (*n* = 12 AC lPVL, *n* = 14 AC hPVL). Wilcoxon signed-rank paired test with Holm correction for multiple comparisons (**C**–**F**, **I**, and **J**); Wilcoxon signed-rank unpaired test for comparisons between UC and AC lPVL, AC lPVL and AC hPVL, and AC hPVL and HAM (**G** and **H**). *P < 0.05, **P < 0.01, ***P < 0.001, ****P < 0.0001.

Given the high proinflammatory cytokine potential of DP T cells, we investigated the contribution of DP T cells to anti–HTLV-1 immunity and inflammation. Peripheral blood mononuclear cells (PBMCs) from ACs were stimulated with heat-inactivated HTLV-1, and HTLV-1–specific CD4^+^ and CD8^+^ SP T cells were identified based on the expression of CD154 or the coexpression of CD69 and CD137, respectively (Figure 4, A and B) (33, 34). The frequencies of CD154^+^ and CD69^+^CD137^+^ cells were higher in DP T cells than CD4^+^ or CD8^+^ SP T cells (Figure 4, C and D). This trend was consistent across UCs and patients with HAM (Figure 4, E and F). The frequency of HTLV-1–specific SP CD4^+^ and CD8^+^ T cells increased with PVL, but there were no significant differences in the frequency of CD154^+^ HTLV-1–specific DP T cells among the different HTLV-1–infected groups (Figure 4, G and H). DP T cells, similarly to CD8^+^ SP T cells, contained a higher frequency of CD69^+^CD137^+^ cells in hPVL groups, and CD4^dim^CD8^+^ DP T cells from patients with HAM expressed significantly higher levels of CD69^+^CD137^+^ than AC hPVL (Figure 4H and Supplemental Figure 4A). Intriguingly, there was no significant difference in the expression of CD154 or CD137 and CD69 by DP T cells stimulated with HTLV-1 or a vehicle-stimulated control (Supplemental Figure 4, B and C), suggesting that the DP T cells may have been recently activated by their cognate antigen in vivo.

In agreement with the results obtained by polyclonal stimulation (Figure 3D), HTLV-1– specific DP T cells displayed a proinflammatory phenotype in response to HTLV-1 stimulation and expressed high levels of IFN-γ, TNF, and IL-17A (Figure 4I). Again, DP T cells expressed levels of granzyme B similar to CD8^+^ SP T cells and levels of IL-21 similar to CD4^+^ SP T cells. In addition to producing high levels of proinflammatory cytokines, DP T cells also displayed increased proliferation measured by Ki67 and increased frequency of CXCL10-producing cells relative to SP T cells after HTLV-1 stimulation (Figure 4J). Overall, these data suggest that DP T cells contain an increased frequency of HTLV-1–specific cells compared with both CD4^+^ and CD8^+^ SP T cells and display a polyfunctional, proinflammatory phenotype.

### HTLV-1–specific DP T cells are phenotypically distinct in ACs and patients with HAM.

**Figure 5:**
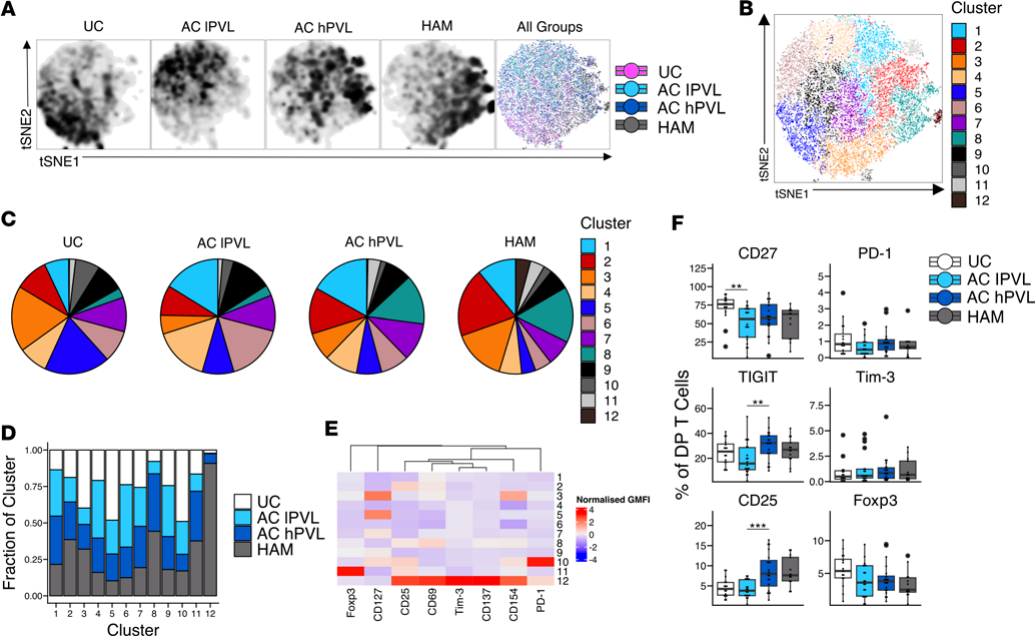
Phenotype of DP T cells in uninfected controls, asymptomatic carriers, and patients with HAM. (**A**) t-SNE plot from a concatenated sample of DP T cells (*n* = 11 each group). (**B**) t-SNE plots depicting the cell clusters identified by Phenograph. (**C**) Pie charts showing the fraction of cells within each cell cluster in uninfected controls or HTLV-1–infected groups. (**D**) Bar graph showing the distribution (fraction) of cells from each patient group in each identified cluster. (**E**) Heatmap of the expression of markers by each cluster displayed as modified *Z* scores using median values. (**F**) Box-and-whisker plots showing the expression of extracellular markers ex vivo in DP T cells from different patient groups (*n* = 16 UC, *n* = 20 AC lPVL, *n* = 20 AC hPVL, *n* = 13 HAM). Wilcoxon signed-rank unpaired test for comparisons between UC and AC lPVL, AC lPVL and AC hPVL, and AC hPVL and HAM (**F**). **P < 0.01, ***P < 0.001.

To determine whether HTLV-1–specific DP T cells were different in UCs, ACs, and patients with HAM, we characterized DP T cells (*n = 11* in each group) after stimulation with heat-inactivated HTLV-1. Dimensionality reduction tools demonstrated that, while there was some overlap in the global phenotype of the 4 groups, HTLV-1–specific DP T cells from these 4 groups were clearly distinct from each other on a t-SNE plot (Figure 5A). To further describe the phenotypic differences, we applied a clustering algorithm using markers of T cell activation (CD154, CD69, CD137), Treg-associated markers (CD25, CD127, Foxp3), and other inhibitory markers (PD-1, Tim-3). Twelve subpopulations of DP T cells were identified based on the expression of these markers (Figure 5B and Supplemental Figure 5A). The size of these subpopulations varied between HTLV-1–infected groups and UCs (Figure 5C). Clusters 3 and 5 were predominant in UCs; clusters 1, 4, and 6 were predominant in AC lPVL; clusters 1, 2, and 8 made up large proportions of the DP T cells in AC hPVL, and a large proportion of HAM DP T cells fell into clusters 2, 3, and 8. Moreover, HAM DP T cells were overrepresented in clusters 2, 3, 8, 11, and 12 and were underrepresented in clusters 1, 4, 5, and 6 (Figure 5D and Supplemental Figure 5B). Cluster 12 was almost entirely composed of HAM DP T cells and was characterized by the expression of high levels of T cell activation markers (CD154, CD137, CD69, and CD25), high levels of Tim-3, and moderate to low expression of Foxp3 and CD127 (Figure 5E). Cluster 5, which consisted mainly of DP T cells from UCs and AC lPVL, was characterized by the expression of high levels of CD127 and low levels of CD69 (Figure 5E). Ex vivo DP T cells from HTLV-1 carriers expressed lower levels of CD27 than UCs, TIGIT was expressed at higher levels on DP T cells from AC hPVL relative to both AC lPVL and HAM, and there was a trend toward increased CD25 expression with increased PVL (Figure 5F).

**Figure 6:**
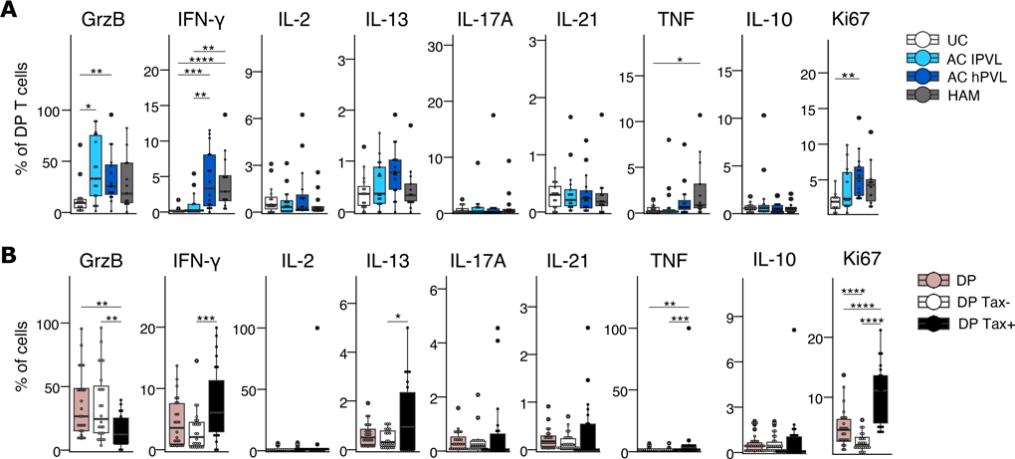
Cytokine expression in DP T cells in response to HTLV-1 stimulation. (**A**) Box-and-whisker plots showing cytokine, Ki67, and granzyme B expression by DP T cells from different patient groups after stimulation with heat inactivated HTLV-1 for 20 hours (*n* = 16 UC, *n* = 17 AC lPVL, *n* = 16 AC hPVL, *n* = 13 HAM). (**B**) Box-and-whisker plots showing cytokine, Ki67, and granzyme B expression by Tax^–^ DP T cells and HTLV-1–infected Tax^+^ DP T cells after stimulation with heat-inactivated HTLV-1 for 20 hours (*n* = 13 AC hPVL, *n* = 11 HAM). Wilcoxon signed-rank unpaired test with Holm correction for multiple comparisons (**A**); Wilcoxon signed-rank paired test with Holm correction for multiple comparisons (**B**). *P < 0.05, **P < 0.01, ***P < 0.001, ****P < 0.0001.

The cytokine expression profiles of DP T cells after stimulation with heat-inactivated HTLV-1 were also compared between groups. DP T cells from AC expressed higher levels of granzyme B than UCs (Figure 6A). hPVL groups expressed higher levels of IFN-γ and TNF than AC lPVL. HAM DP T cells produced more TNF than ACs and UCs, but the difference was only statistically significant with UCs. There were no significant differences in the levels of IL-2, IL-13, IL-17A, IL-21, and IL-10 expression between HTLV-1–infected groups and UCs. DP T cells from HTLV-1 carriers had increased proliferation compared with UCs as measured by Ki67 expression. These data suggest that HTLV-1–specific DP T cells are phenotypically different among HTLV-1–infected groups but display a similar capacity to secrete proinflammatory cytokines and cytotoxic molecules.

To determine whether the proinflammatory cytotoxic helper phenotype was due to HTLV-1–infected DP T cells, –uninfected DP T cells, or both, we used Tax expression in hPVL groups to identify HTLV-1–infected DP T cells and characterized the cytokine expression profile of Tax^+^ and Tax^–^ DP T cells after stimulation with heat-inactivated HTLV-1 (Figure 6B). There was no difference in the expression of IL-2, IL-17A, IL-21, and IL-10 between Tax^+^ and Tax^–^ DP T cells. Tax^+^ DP cells expressed less granzyme B than DP Tax^–^ cells and more IFN-γ, IL-13, TNF, and Ki67. Despite Tax^+^ DP T cells expressing less granzyme B than Tax^–^ DP T cells, they were still significantly more cytotoxic than CD4^+^ SP T cells. Overall, both Tax^+^ and Tax^–^ DP T cells were highly proinflammatory when stimulated with HTLV-1 and expressed high levels of IL-21 and other helper cytokines, but Tax^+^ DP T cells appeared to be more proinflammatory and less cytotoxic than Tax^–^ DP T cells.

### DP T cells induce proinflammatory astrocytes.

**Figure 7:**
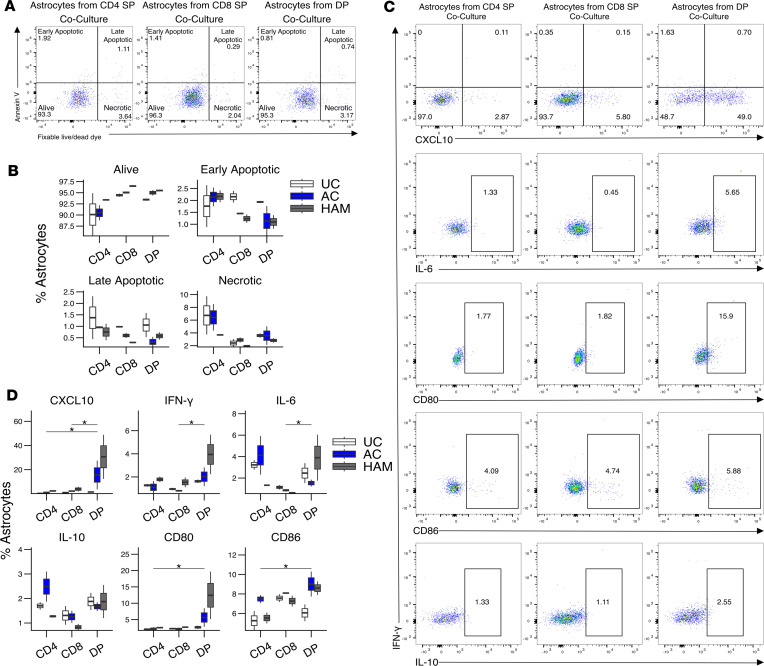
DP T cells induce an inflammatory phenotype in astrocytes. (**A** and **B**) Representative flow plot (**A**) and box-and-whisker plot (**B**) showing alive, early apoptotic, late apoptotic, and necrotic astrocytes after 36 hours of coculture with HTLV-1 prestimulated CD4^+^ SP, CD8^+^ SP, or DP T cells (*n* = 2 UC, *n* = 2 AC, *n* = 2 HAM). (**C** and **D**) Representative example (**C**) and summary box-and-whisker plot (**D**) showing the expression of extracellular markers, cytokines, and chemokines in astrocytes cocultured with HTLV-1–stimulated CD4^+^ SP, CD8^+^ SP, or DP T cells (*n* = 2 UC, *n* = 2 AC, *n* = 2 HAM). Wilcoxon signed-rank paired test for comparisons between CD4^+^ SP and DP or CD8^+^ SP and DP (**B** and **D**). *P < 0.05.

Given the increased migratory potential of DP T cells due to high CD49d and CXCR3 expression, their proinflammatory cytotoxic helper phenotype, and their increased frequency in patients with HAM, we wanted to explore the role that DP T cells may play in HAM CNS pathology. For this, we set up cocultures of HTLV-1–stimulated DP T cells isolated from UCs, AC, and patients with HAM with an astrocyte cell line for 36 hours. There were no significant differences in the viability of the astrocytes in the different coculture conditions (Figure 7, A and B). However, DP T cells induced a significant increase in the expression of CXCL10, IFN-γ, CD80, and CD86, especially in those astrocytes cocultured with DP T cells isolated from patients with HAM (Figure 7, C and D). Astrocytes cocultured with DP T cells expressed significantly more IL-6 than those cocultured with CD8^+^ SP T cells but not than those cocultured with CD4^+^ SP T cells. These data indicate that DP T cells, and particularly those from patients with HAM, induce proinflammatory astrocytes that have the potential to contribute to CNS pathology.

### HTLV-1 infection of SP T cells gives rise to a DP T cell population.

**Figure 8:**
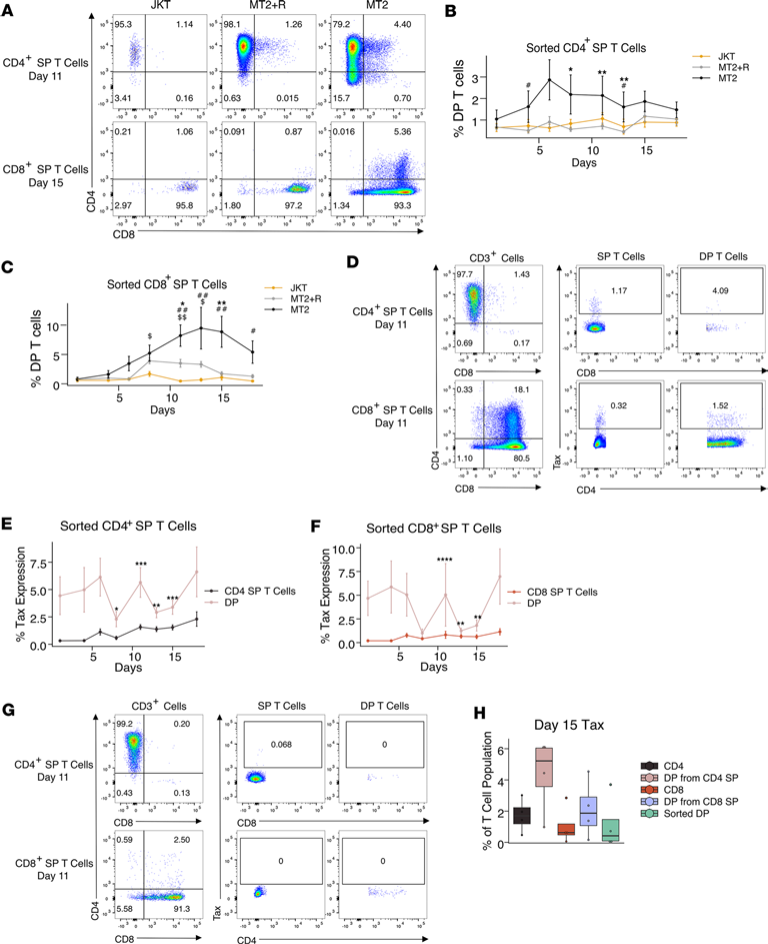
Origin of DP T cells in HTLV-1 infection. (**A**) Representative plot showing CD4 and CD8 expression on sorted CD4^+^ SP and CD8^+^ SP T cells after coculture with JKT, MT2+R, or MT2 cells. (**B** and **C**) Line graphs showing the frequency of emerged DP T cells during coculture of SP T cells with JKT, MT2+R, or MT2 cells. (**D** and **G**) Representative dot plots showing CD4 and CD8 expression and Tax expression in SP T cells and DP T cells during coculture with MT2 cells (**D**) or MT2+R (**G**). (**E** and **F**) Line graphs showing Tax expression by SP T cells and the emerged DP T cells during coculture with MT2 cells. (**H**) Box-and-whisker plot showing Tax expression in CD4^+^ SP T cells, emerged DP T cells from CD4^+^ SP T cells, CD8^+^ SP T cells, emerged DP T cells from CD8^+^ SP T cells, and naturally occurring DP T cells on day 15 of coculture with MT2 cells (*n* = 4). Data are shown as mean ± SEM (*n* = 15) (**B**, **C**, **E**, and **F**). Wilcoxon signed-rank unpaired test was used. *P < 0.05, **P < 0.01 for comparisons between MT2 and MT2+R; ^#^*P* < 0.05, ^##^*P* < 0.01 for comparisons between MT2 and JKT; ^$^*P* < 0.05, ^$$^*P* < 0.01, for comparisons between MT2+R and JKT (**B** and **C**). Wilcoxon signed-rank paired test was used. *P < 0.05, **P < 0.01, ***P < 0.001 for comparisons between SP T cells and DP T cells at each time point (**E** and **F**).

Given that the increased frequency of DP T cells in patients with HAM compared with ACs did not seem to be due to increased proliferation (Figure 6A), we questioned how this DP T cell population arose. To determine whether HTLV-1 infection was involved in the generation of DP T cells, we infected flow-sorted CD4^+^ and CD8^+^ SP T cells from uninfected individuals with HTLV-1 by coculturing them with irradiated MT2 cells, a leukemic HTLV-1–infected cell line (35). CD4^+^ and CD8^+^ SP T cells were also cocultured with irradiated MT2 cells in the presence of raltegravir, a HIV integrase inhibitor that inhibits HTLV-1 integration in vitro (36), and with the non–HTLV-1–infected leukemic cell line Jurkat (JKT) as controls. SP T cells cocultured with MT2 cells and raltegravir (MT2+R) were therefore exposed to HTLV-1 virions/proteins and other molecules secreted by MT2 cells but were not stably infected by HTLV-1 (37, 38), and JKT cells were used to control for long-term in vitro culture and exposure to foreign non–HTLV-1 proteins and other secreted molecules (39). By measuring CD4 and CD8 expression every 2–3 days in the sorted SP T cell populations, we observed the emergence of DP T cell populations from both the sorted CD4^+^ and CD8^+^ SP T cells cocultured with MT2 cells, but not from the JKT controls (Figure 8, A–C). A small DP T cell population appeared to emerge from CD8^+^ SP T cells cocultured with MT2+R, but the DP T cell population is significantly smaller than that induced by the MT2 cell coculture and is shorter lived (Figure 8C). Most of the DP T cells that emerged from CD4^+^ and CD8^+^ SP T cells were CD4^+^CD8^dim^ and CD4^dim^CD8^+^, respectively (Figure 8A). DP T cell frequency peaked between days 6 and 11 in the sorted CD4^+^ SP T cells and between days 11 and 15 for the sorted CD8^+^ SP T cells. The DP T cell population that emerged from CD8^+^ SP T cells was larger than that derived from CD4^+^ SP T cells.

We measured Tax expression in the cultured cells to track HTLV-1 infection (Figure 8D). DP T cells derived from both CD4^+^ and CD8^+^ SP T cells expressed higher levels of Tax than their parent SP T cell populations, indicating higher rates of infection in the newly generated DP T cells (Figure 8, E and F) and consistent with the increased ex vivo infection rate of DP T cells (Figure 1D). There was virtually no Tax expression in cells that were cocultured in the presence of raltegravir, confirming that HTLV-1 was not able to productively infect these cells (Figure 8G). Sorted, naturally occurring DP T cells from UCs cocultured with MT2 cells expressed levels of Tax similar to those of SP T cells cocultured with MT2 cells and expressed less Tax than the DP T cells that emerged from the SP T cells (Figure 8H), suggesting that naturally occurring DP T cells from UCs are not preferentially infected with HTLV-1 in vitro and supporting the notion that the high level of DP T cell infection is due to HTLV-1–infected SP T cells differentiating into DP T cells.

**Figure 9:**
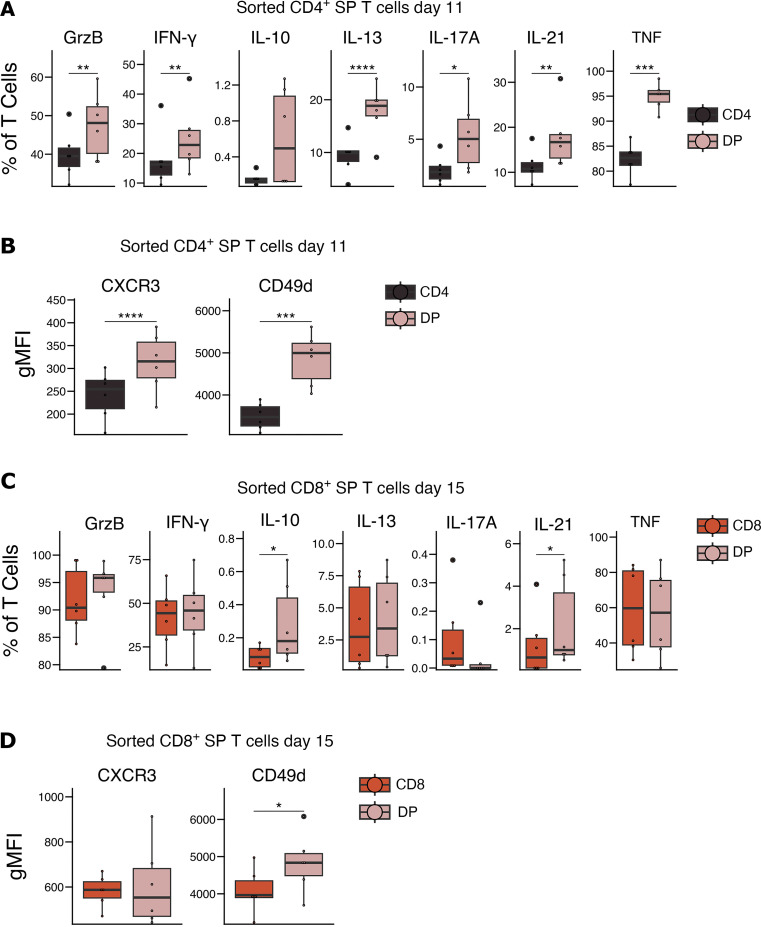
Characterization of the emerged DP T cell population after HTLV-1 infection. (**A**–**D**) Box-and-whisker plots showing cytokines and granzyme B expression, and the gMFI of extracellular markers in SP T cells and the newly emerged DP T cells from CD4^+^ SP T cells (**A** and **B**) and CD8^+^ SP T cells (**C** and **D**) after coculture with MT2 cells, after stimulation with PMA and ionomycin (*n* = 6). Wilcoxon signed-rank paired test (**A**–**C**) and paired *t* test (**D**). *P < 0.05, **P < 0.01, ***P < 0.001, ****P < 0.0001 for comparisons between SP T cells and DP T cells.

To assess whether the in vitro–generated DP T cells reflected the phenotype of naturally occurring DP T cells in HTLV-1 carriers, we characterized the cytokine expression profiles and extracellular markers in the emerged DP T cell population. We stimulated the cells with PMA and ionomycin for 4 hours after 11 or 15 days of coculture of the sorted CD4^+^ or CD8^+^ SP T cells with MT2 cells, respectively, and compared the phenotype of the emerged DP T cells with the remaining parent SP T cell population. The DP T cells that emerged from the CD4^+^ SP T cells upregulated granzyme B and became more proinflammatory that their SP T cell counterparts, expressing significantly higher levels of IFN-γ, IL-13, IL-17A, IL-21, and TNF (Figure 9A). We also observed an increase in CXCR3 and CD49d expression in the DP T cells when compared with CD4^+^ SP T cells (Figure 9B). The DP T cells that emerged from CD8^+^ SP T cells expressed higher levels of IL-21 than their parent population (Figure 9C) and increased expression of IL-10 and CD49d, but there was no significant change in the expression of granzyme B, IFN-γ, IL-13, IL-17A, TNF, or CXCR3 (Figure 9, C and D). These data are consistent with our ex vivo observations where DP T cells expressed comparable levels of granzyme B and IFN-γ as CD8^+^ SP T cells and levels higher than CD4^+^ SP T cells; they expressed lower levels of CXCR3 than CD8^+^ SP T cells and higher levels than CD4^+^ SP T cells, and they expressed higher levels of IL-13, IL-17A, IL-21, TNF, and CD49d than either SP T population (Figure 3, C and D).

### Involvement of ThPOK and RunX3 in DP T cell generation and stability.

**Figure 10:**
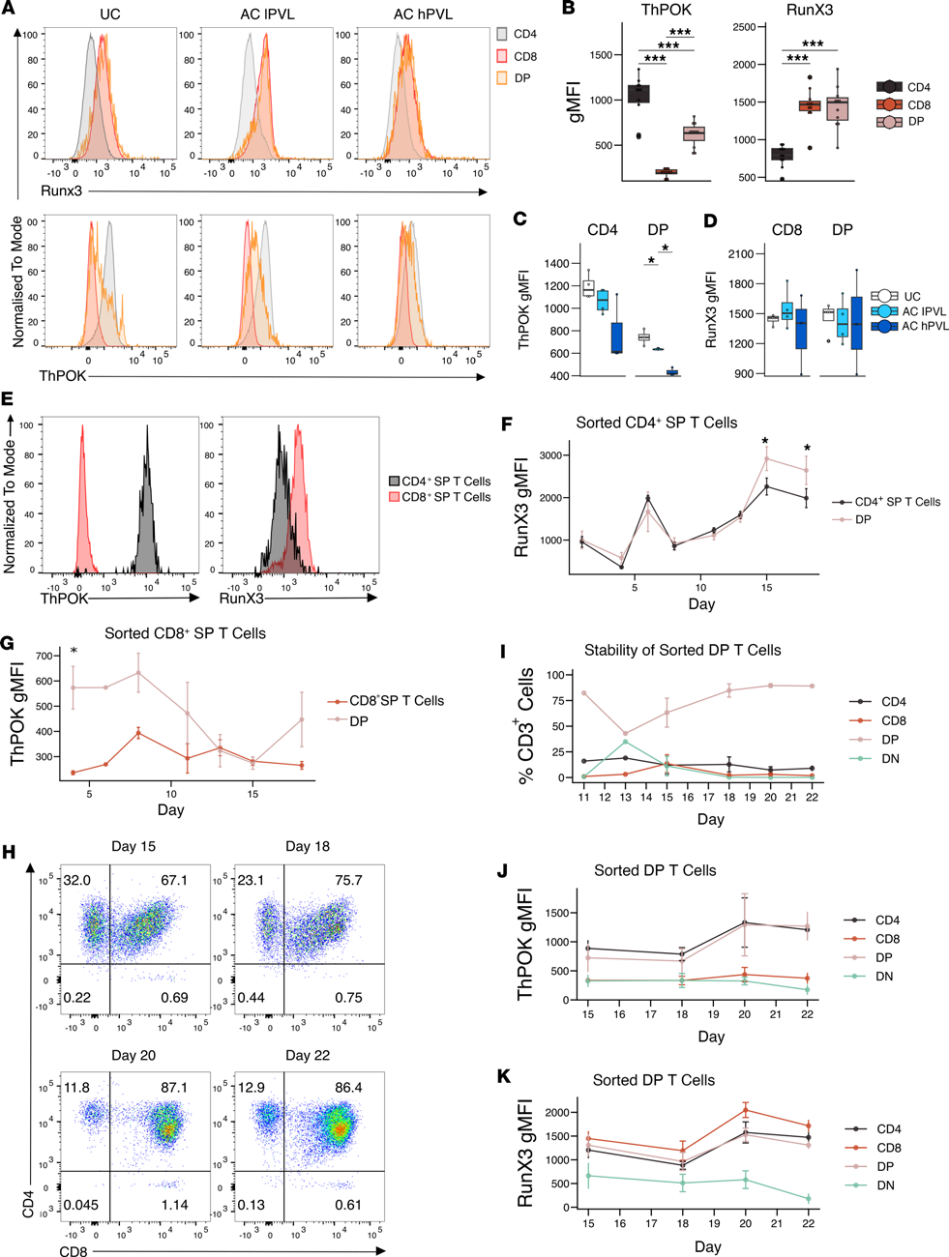
Involvement of ThPOK and RunX3 in CD4 and CD8 coexpression. (**A** and **B**) Representative examples (**A**) and summary gMFI (**B**) in UC, AC lPVL, and AC hPVL of ex vivo Runx3 and ThPOK expression in CD4^+^ SP, CD8^+^ SP, and DP T cells (*n* = 11). (**C** and **D**) Box-and-whisker plots showing ThPOK and RunX3 gMFI in different patient groups (*n = 4* UC*, n = 4* AC lPVL*, n = 3* AC hPVL). (**E**) Histogram showing the expression of RunX3 and ThPOK in sorted CD4^+^ and CD8^+^ SP T cells on day 1 of coculture with MT2 cells. (**F** and **G**) Line graph showing RunX3 and ThPOK gMFI in emerged DP T cells and CD4^+^ SP or CD8^+^ T cells during coculture with MT2 cells; data are shown as mean ± SEM (*n* = 5). (**H** and **I**) Representative dot plot (**H**) and line graph (**I**) showing the percentage of CD4^+^ SP, CD8^+^ SP, DP, and DN T cells that emerge from sorted naturally occurring DP T cells cocultured with MT2 cells; data are shown as mean ± SEM (*n* = 3). (**J** and **K**) Line graphs showing ThPOK and RunX3 gMFI in CD4^+^ SP, CD8^+^ SP, DP, and DN T cell populations that emerged from sorted naturally occurring DP T cells cocultured with MT2 cells; data are shown as mean ± SEM (*n* = 3). Wilcoxon signed-rank paired test with Holm correction (**B**); Wilcoxon signed-rank unpaired test for comparisons between UC and AC lPVL, and AC lPVL and AC hPVL (**C** and **D**); paired *t* test for comparisons between SP and DP T cells at each time point (**F** and **G**). *P < 0.05, ***P < 0.001.

Little is known about how peripheral DP T cells emerge in UCs or during chronic viral infections. The transcription factors RunX3 and ThPOK have been shown to regulate thymic CD8^+^ and CD4^+^ T cell lineage commitment, respectively, and are thought to be mutually exclusively expressed in naive T cells (40–42). Ex vivo DP T cells from UCs and ACs expressed an intermediate level of ThPOK relative to CD8^+^ and CD4^+^ SP T cells and expressed comparable Runx3 levels to CD8^+^ SP T cells (Figure 10, A and B). Interestingly, both CD4^+^ SP and DP T cells from AC hPVL expressed lower levels of ThPOK as compared with AC lPVL, who in turn expressed lower levels than UCs (Figure 10C). There was no difference in RunX3 expression between patient groups (Figure 10D). We observed a significant negative correlation between PVL and ThPOK gMFI in CD4^+^ and DP T cells (Supplemental Figure 6A), and while the trend was similar for RunX3 in DP T cells, it did not reach statistical significance (Supplemental Figure 6B).

While the expression of ThPOK and RunX3 are thought to be mutually exclusive in naive T cells, we decided to examine the expression of both transcription factors in SP T cells, as they were cocultured with MT2 cells to induce DP T cells. Sorted CD4^+^ and CD8^+^ SP T cells expressed high levels of ThPOK and RunX3, respectively, after 24 hours in coculture with MT2 cells (Figure 10E), but both RunX3 and ThPOK levels varied during the 18-day coculture with MT2 cells (Figure 10, F and G). Significantly increased expression of RunX3 in the emerging DP T cell population compared with the parent CD4^+^ T cell population was observed at later time points (Figure 10F). In the sorted CD8^+^ SP T cell culture, the emerged DP T cell population expressed significantly higher ThPOK levels compared with CD8^+^ SP T cells, which gradually became more similar to those of the bulk CD8^+^ SP T cell population over time (Figure 10G).

The expression of CD4 and CD8 in the sorted, naturally occurring DP T cells from UCs was not stable over time when cocultured in vitro with MT2 cells (Figure 10, H and I). While the majority of sorted DP T cells remained DP, SP and DN T cell populations emerged (Figure 10, H and I). The emerged CD8^+^ SP and DN T cells downregulated ThPOK but not the CD4^+^ SP T cell population (Figure 10J). RunX3 was upregulated by the CD8^+^ SP T cell population that emerged and was downregulated by the DN population (Figure 10K). These results suggest that ThPOK is associated with CD4^+^ expression in SP T cells and that RunX3 is associated with CD8^+^ SP T cells. However, ThPOK and RunX3 expression appear to be dynamic and were not always correlated with CD4^+^ or CD8^+^ expression in DP T cells. It is likely that other transcription factors and/or HTLV-1 proteins are involved in DP T cell differentiation in infected cells.

## Discussion

Here we show that DP T cells in HTLV-1 carriers are highly infected, are more abundant in patients with HAM, and are proinflammatory with a helper/cytotoxic phenotype. These DP T cells express high levels of CD49d and CXCR3, suggesting an enhanced capacity to migrate to the CNS, and they are able to induce proinflammatory astrocytes demonstrating their potential to contribute to HAM pathology. Mechanistically, we show that HTLV-1–infected DP T cell populations are generated by infection of SP CD4^+^ and CD8^+^ T cells. The increased migratory capacity and proinflammatory phenotype of DP T cells and their ability to induce proinflammatory astrocytes suggest that they could contribute to CNS pathology.

DP T cells in HTLV-1–infected individuals are heterogeneous, but they all display a highly inflammatory phenotype with high levels of activation-induced markers and proinflammatory cytokines. The coexpression of helper cytokines, granzyme B, and CD154 suggests that DP T cells can act as helper cells, providing help to both B and CD8^+^ T cells (43), but that they can also perform cytotoxic functions that are potentially detrimental in patients with HAM. Approximately half of DP T cells in AC are antigen experienced, as shown by the downregulation of CD27. While HTLV-1 infection has been shown to increase Foxp3^+^ expression, higher Foxp3 expression in DP T cells relative to CD8^+^ SP T cells could be indicative of an activated nonregulatory phenotype (44–46). Despite this proinflammatory effector phenotype, DP T cells also expressed high levels of inhibitory receptors PD-1, Tim-3, and TIGIT. High levels of inhibitory receptor expression in chronic viral infections are often associated with T cell exhaustion (47). However, DP T cells in people infected with HTLV-1 are still able to proliferate at a higher rate than SP T cell populations when stimulated with HTLV-1 and have a higher cytokine secretion potential. This observation, in addition to the high levels of activation-induced marker expression observed, suggests that DP T cells are an active effector T cell population in HTLV-1 infection.

The increased frequency of highly inflammatory DP T cells in patients with HAM may contribute to tissue damage in the CNS. CD4^+^CD8^+^ T cells in patients with HAM were infected at higher rates, and CD4^dim^CD8^+^ T cells expressed significantly more CD69 and CD137 than AC hPVL. Expanded DP T cell populations have been associated with other inflammatory diseases, including MS and RA, as well as in viral infections such as HIV, HCV, and EBV (19–24), but it is unclear if these expanded DP T cell populations cause inflammation or arise as a consequence of inflammation. It is likely that both scenarios occur in vivo. DP T cells induced proinflammatory astrocytes, causing them to secrete high levels of CXC10, IFN-γ, and IL-6 and upregulate CD80 and CD86. Our results support the hypothesis that inflammatory DP T cells in HTLV-1 infection can contribute to HAM pathogenesis. Indeed, DP T cell in HTLV-1 carriers express high levels of molecules involved in T cell migration to tissues, including CXCR3 and CD49d. CD49d is a major integrin that allows activated T cells to cross the blood-brain barrier (BBB), and antibodies blocking VLA-4 represent a current MS treatment (24, 48, 49). In agreement with this, DP T cells have been shown to be enriched in the CSF relative to the periphery of patients with MS and patients with noninflammatory neurological diseases (24). In addition, DP T cells are able to induce proinflammatory astrocytes that express high levels of CXCL10, which chemoattracts CXCR3-expressing cells. CXCR3 expression is typically associated with Th1 CD4^+^ T cells, cytotoxic CD8^+^ T cells, and DP T cells (50–53), and it has been shown to activate peripheral immune cells, microglia, and CNS resident macrophages during brain injury to direct them to the lesion site (51, 52, 54–57). CXCL9 and CXCL10 are found at higher concentrations in the CSF of patients with HAM than in patients with MS, who in turn have significantly higher levels than healthy controls. Moreover, the main source of CXCL10 in the CSF of patients with HAM is thought to be astrocytes (58). Despite DP T cells being enriched in the CSF of patients with MS, DP T cells did not appear to be present in active MS lesions from autopsy material from patients with MS (58). This is consistent with our in vitro data, in which we did not observe a decrease in astrocyte viability during the coculture with DP T cells but in which we did observe changes to the astrocyte phenotype. DP T cells in HTLV-1 carriers also expressed high levels of activation induced markers CD154, CD137, and CD69 in response to HTLV-1 stimulation, and CD49d^+^CD154^+^ lymphocytes have been shown to reprogram oligodendrocytes into immune reactive cells in MS (59), further supporting the potential for DP cells in HTLV-1 infection to contribute to CNS pathology. In addition, the high HTLV-1 infection rate of DP T cells also provides a potential source of antigen for local reactivation of T cells once in the CNS. These results warrant further studies on the role DP T cells play in recruiting other T cells to the CNS, their contribution to inflammation in the CNS, and their role in CNS pathology.

Our data suggest that the majority of infected DP T cells arise from SP T cells, in agreement with works demonstrating that CD4^+^CD8^+^ T cells in an HIV-infected person share their TCR sequence with CD4^+^ SP T cells (18, 60). In addition, excess IL-4 has been shown to induce CD8 expression on CD4^+^ SP T cells but not CD8^+^ SP T cells (61), and CD4^dim^CD8^+^ T cells have previously been described as an activated CD8^+^ T cell phenotype (62). Moreover, mature antigen-stimulated CD4^+^ T cells have been shown to terminate the expression of ThPOK and activate the expression of genes of the CD8^+^ T cell lineage, resulting in cytotoxic CD4^+^CD8^+^ T cells that were MHC class II restricted (63). Our results demonstrate that HTLV-1 infection drives the emergence of a DP T cell population from both SP T cell population and that DP T cell population is highly HTLV-1 infected. It is likely that high proliferation rates of infected DP T cells contribute to expanding the HTLV-1–infected DP T cell population in addition to the de novo HTLV-1 infection. The DP T cell population that emerged during MT2 coculture of SP T cells from UCs appeared to be partially stable, and whether this is the case for in vivo–generated DP T cells in HTLV-1 carriers remains to be determined. Our data suggest that de novo HTLV-1 infection of SP T cells results in the coexpression of CD4 and CD8 in a subset of infected cells and correlates with ThPOK and RunX3 expression, but other transcription factors are likely involved in the reacquisition of CD4 or CD8 expression. The underlying mechanisms of how HTLV-1 induces this population warrant further investigations: possibilities include the specific location of the HTLV-1 integration site in the host cell genome, the cytokine milieu, and the activity of HTLV-1 proteins.

Several questions emerge from this work, including elucidation of whether these DP T cells are MHC class I or class II restricted, molecular mechanisms by which HTLV-1 infection and viral proteins induce the DP T cell population, and identification of integration sites of HTLV-1–infected DP- and SP-infected T cells to determine if the integration site locations drive the coexpression of CD4 and CD8. Follow-up studies on the role and generation of DP T cells in HTLV-1 infection are warranted to answer these questions.

## Methods

### Participants, study design, and clinical data collection.

HTLV-1–infected individuals attended the National Centre for Human Retrovirology, Imperial College Healthcare NHS Trust, at St. Mary’s Hospital, London, United Kingdom. HTLV-1 infection was diagnosed by the Virus Reference Division of UK Healthy Security Agency using commercially available enzyme-linked immunoassays and confirmed using Western blot (Genelabs, HTLV 2.4) according to the manufacturer’s instructions. The diagnosis of HAM was made using the World Health Organization (WHO) criteria, and patients had not been on treatment for at least 3 months before blood donation. Uninfected volunteers were Imperial College staff members. The UCs, ACs, and patients with HAM were matched on age and biological sex (Supplemental Table 1).

### Cell isolation, storage, and thawing.

Peripheral blood was collected in sodium heparin tubes, and PBMCs were isolated immediately (<2 hours) after blood draw by Ficoll Hypaque gradient. The PBMC were either used fresh or stored at –150°C. After thawing, PBMCs were rested for 2 hours in media: RPMI 1640 (Thermo Fisher Scientific, 21875034) media supplemented with 2 mM L-Glutamine, 5% human serum (HS; MilliporeSigma, lot SLBX6359), and 100 μg/mL penicillin and streptomycin (Thermo Fisher Scientific, 15140-122) before experiments.

### Cell staining for flow cytometry or fixed cell sorting.

For ex vivo phenotypic characterization, 500,000–2 million PBMCs were stained after resting. During T cell and MT2/JKT cocultures, 50,000–200,000 cells were stained at each time point. For all stainings, cells were stained with LIVE/DEAD Fixable Dead Cell Dyes (Thermo Fisher Scientific, L23105) according to the manufacturer’s specifications. An Fc receptor (FcR) blocking step was performed using FcR Blocking Reagent Human (Miltenyi Biotec) before cell surface antibody staining. The antibodies used in the cell surface staining were the following: CD3 (UCHT1), CD4 (RPA-4), CD25 (M-A251), CXCR3 (G025H7), CD80 (2D10), CD86 (BU63), CD49d (9F10), CD8 (SK1), CD27 (0323), and CD137 (4B4-1) from BioLegend; CD154 (24-31, Invitrogen); CD69 (FN50, Invitrogen); CD45RA (HI100, BD Biosciences); Tim-3 (F38-2E2, Invitrogen); CD127 (hIL-7R-M21, BD Biosciences); PD-1 (MIH4, BD Biosciences; EG12.2H7 BioLegend); CCR7 (3D12, eBioscience); and TIGIT (MBSA43, Invitrogen). Cells were subsequently fixed using the Foxp3 staining buffer kit (Invitrogen, 00-5523-00) following the manufacturer’s specifications. Cells were stained intracellularly after fixation. The antibodies used for intracellular staining were the following: IL-17A (TC11-18H10.1), IL-17A (BL168), IL-10 (JES3-19F1), CXCL10 (J034D6), IL-6 (MQ2-13A5), GM-CSF (BVD2-21C11), ThPOK (11H11A14), TNF (Mab11), IFN-γ (4S.B3), and IL-13 (JES10-5A2) from BioLegend; IL-21 (eBio3A3-N2, Invitrogen); granzyme B (GB11, BD Biosciences); Ki67 (B56, BD Biosciences); L-2 (MQ1-17H12, BD Biosciences); Foxp3 (PCH 101, Invitrogen); RunX3 (R3-5GH Invitrogen); and Tax (LT-4) (64). Intracellular staining was performed using the Foxp3 staining buffer kit. Following the staining, cells were resuspended in PBS for flow cytometry analysis.

### Flow cytometry data analysis, dimensionality reduction, and clustering.

Samples were run on a Fortessa instrument (BD Biosciences) and analyzed using FlowJo v.10. To make comparisons between CD4^+^ SP, CD8^+^ SP, and DP T cells ex vivo, dimensionality reduction and t-SNE plots were obtained by downsampling 14 AC lPVL and 14 AC hPVL samples to 416 cells per T cell population per sample. The concatenated sample was used to calculate t-SNE axes using 1,000 iterations, perplexity of 30, and the default learning rate of 2446. The following compensated parameters were included in the t-SNE analysis: Tim-3, CD45RA, CD27, FoxP3, PD-1, CCR7, TIGIT, CD25, and CD127. To make comparisons between the DP T cell populations of different patient groups, dimensionality reduction and t-SNE plots were obtained by downsampling 11 samples per group (UC, AC lPVL, AC hPVL, HAM) to 523 DP T cells per sample, and the concatenated sample was used to calculate t-SNE axes using 1,000 iterations, perplexity of 30, and the default learning rate of 1610. In order to identify cell clusters, we used the Phenograph plugin in FlowJo, with *k* = 137 and the following compensated parameters: CD154, CD25, Tim-3, CD137, CD69, FoxP3, PD-1, and CD127.

### Generation of heat-inactivated HTLV-1.

The HTLV-1–producing MT2 cell line was cultured in complete T cell media — RPMI 1640 media; Thermo Fisher Scientific, 21875034) supplemented with 5% HS, 10 mM Hepes buffer solution (Thermo Fisher Scientific, 15630-056), 1× nonessential amino acids (Thermo Fisher Scientific, 11140), 1 mM sodium pyruvate (Thermo Fisher Scientific, 11360-070), 2 mM L-Glutamine (Thermo Fisher Scientific, 25030-024), and 100 μg/mL penicillin and streptomycin (Thermo Fisher Scientific, 15140-122) — at 0.3 million cells/mL for 7 days. The cell culture media containing the HTLV-1 virions were collected and centrifuged for 15 minutes at 400*g* at 4°C to remove large debris. Three volumes of supernatant were mixed with 1 volume of Lenti-X concentrator (Takara Bio, 631231) and incubated at 4°C for 24 hours, followed by centrifugation at 1,500*g* for 45 minutes at 4°C. The pellet was resuspended in 1/100 of the initial volume of media with PBS and was heat-inactivated by incubation at 56°C for 4 hours, aliquoted, and stored at –80°C. The concentration of HTLV-1 p19 was quantified before heat inactivation using a p19 ELISA kit (ZeptoMetrix, 080116).

### Ex vivo stimulation assays.

After resting, PBMCs were stimulated for 12–20 hours at 10 million cells/mL with heat-inactivated HTLV-1 at a concentration of 0.92 μg/mL p19, 1 μg/mL anti-CD40 (Miltenyi Biotec, 130-094-133), and 1 μg/mL anti-CD28 (Miltenyi Biotec, 130-093-975). For intracellular stainings, 3 μg/mL GolgiStop^T^ and 1 μg/mL GolgiPlug (BD Biosciences) were added to the cultures 11 hours after the start of the stimulation for an additional 9 hours.

For PMA and ionomycin stimulation experiments, T cells were stimulated with 50 ng/mL PMA, 250 ng/mL ionomycin, 3 μg/mL GolgiStop, and 1 μg/mL GolgiPlug (BD Biosciences) for 4 hours.

### Sorting of fixed cells for ddPCR.

Total PBMCs were stimulated with heat-inactivated HTLV-1 as previously described for 12 hours. Cells were stained with the following cell surface receptor antibodies: CD3 (UCHT1 and BioLegend), CD4 (RPA-4, BioLegend and BD Biosciences), CD8 (SK1, BioLegend, BD Biosciences), CD69 (FN50, Invitrogen), CD137 (4B4-1, BioLegend), and CD154 (24-31, Invitrogen). Tax was stained after fixation with an anti-Tax antibody (LT-4). The fixed samples were kept on ice and sorted cold. The following gating strategy was used to sort CD4^+^ SP, CD8^+^ SP, DP, and DN T cells: lymphocyte gating based on size, granularity based on FSC versus SSC, and single cells based on FSC-A versus FSC-H and live cells (cells negative for the fixable viability dye). CD4^+^ SP T cells were CD3^+^CD4^+^CD8^–^, CD8^+^ SP T cells were CD3^+^CD4^–^CD8^+^, DP T cells were CD3^+^CD4^+^CD8^+^, and DN cells were CD3^+^CD4^–^CD8^–^. DNA was isolated immediately after sorting. Postsort purity was determined in all samples by flow cytometry and was above 95% in all samples.

### DNA isolation.

DNA was extracted from up to 5 million cells using an optimized protocol for fixed cells (65).

### PVL quantification.

The PVL in the sorted fixed T cell populations was quantified using ddPCR. HTLV-1 primers and probes were designed to bind HTLV-1 *tax,* and *albumin* primers and probes were adapted from previous publications (66). Primers and probes were obtained from Integrated DNA Technologies. A reaction mixture with a final concentration of 2 ng/μL DNA, 900 nm *tax* forward and reverse primers, 600 nM *albumin* forward and reverse primers, 250 nM *tax* probe, 250 nM *albumin* probe, and 1× ddPCR Supermix for Probes (no dUTP, Bio-Rad, 186-3023) was prepared in a total volume of 22 μL. The primer and probes sequences are as follows: HTLV-1 *tax* forward primer: 5′-CGG ATA CCC AGT CTA CGT GTT-3′ (67), HTLV-1 *tax* reverse primer: 5′-CAG TAG GGC GTG ACG ATG TA-3′, HTLV-1 *tax* doble-quencher probe: 5′-56-FAM/CTG TGT ACA/ZEN/AGG CGA CTG CC/3IABkFQ-3′; *albumin* forward primer: 5′-TGC ATG AGA AAA CGC CAG TAA-3′ (66), *albumin* reverse primer: 5′-ATG GTC GCC TGT TCA CCA A-3′ (66), *albumin* doble quencher probe: 5′-/HEX/TGA CAG AGT / ZEN/ CAC CAA ATG CTG CAC AGA A/3lABkFQ/-3′ (66).

The ddPCR reaction was prepared in a 96-well PCR plate, and 20 μL were transferred to an 8-channel DG8 cartridge (Bio-Rad, 1864008) with 70 μL droplet generation oil (Bio-Rad, 1863005). A DG Gasket (Bio-Rad, 1863009) was placed over the cartridge, and the cartridge was placed in the QX200 droplet generator (Bio-Rad, 1864002) for droplet formation. In total, 40 μL of droplets was transferred to a semiskirted clear 96-well PCR plate (Bio-Rad, 12001925), sealed with a pierceable foil heat seal (Bio-Rad, 1814040) in the PX1 PCR Plate Sealer (Bio-Rad, 1814000), and placed into the C1000 Touch Thermal Cycler (Bio-Rad) for amplification. The cycling conditions were as follows: 10 minutes at 95°C, 40 cycles each of 30 seconds at 94°C followed by 1 minute at 60°C, and 10 minutes at 98°C. A 2°C/second ramp rate was set for each cycling step. Samples were transferred immediately to the QX200 Droplet Reader (Bio-Rad). Results from the QX200 droplet reader were recorded in Bio-Rad’s QuantaSoft software (version 1.7) using absolute quantification. The software counts the number of positive and negative droplets and calculates the number of copies/L. The HTLV-1 PVL was calculated by dividing the number of *tax^+^* droplets by the number of *albumin^+^* droplets divided by 2 (to account for the 2 copies of *albumin* in the genome). Each ddPCR experiment had nontemplate negative controls and a positive control with a known HTLV-1 PVL of 100%.

### Ex vivo T cell sorting.

Fresh or thawed and rested PBMCs were stained with the following extracellular antibodies: CD3 (UCHT1, BioLegend), CD4^+^ (RPA-4, BioLegend and BD Biosciences), CD8^+^ (SK1, BioLegend and BD Biosciences). Samples were kept on ice and sorted cold. In total, 1 μg/mL propidium iodide (BD Biosciences) was added to the samples right before the start of the sort to exclude dead cells. The following gating strategy was used to sort CD4^+^ SP, CD8^+^ SP, DP, and DN T cells: lymphocyte gating based on size and granularity-based on FSC versus SSC, single cells based on FSC-A versus FSC-H, and live cells (cells negative for PI). CD4^+^ SP T cells were CD3^+^CD4^+^CD8^–^, CD8^+^ SP T cells were CD3^+^CD4^–^CD8^+^, DP T cells were CD3^+^CD4^+^CD8^+^, and DN cells were CD3^+^CD4^–^CD8^–^. Post-sort purity was determined in all samples by flow cytometry and was above 95% in all samples.

### HTLV-1 infection of sorted T cell populations.

MT2 and JKT cells were cultured for at least 2 weeks prior to experiments at 0.3 million cells/mL in complete T cell media and split once a week until use. Fresh PBMCs from uninfected donors were sorted into CD4^+^ SP, CD8^+^ SP, and DP T cell populations. For T cells cocultured with MT2+R, the sorted T cells were pretreated with 10 μM raltegravir in complete T cell media for 2 hours before the addition of MT2 cells. The sorted T cells were cocultured with either MT2 or JKT cells at a 4:1 ratio at 1 million cells/ mL in complete T cell media supplemented with 20 U IL-2 and, for the raltegravir coculture samples, with 10 μM raltegravir. MT2 and JKT cells had been irradiated with 400 gray to inhibit proliferation and labeled with CellTace CSFE (Thermo Fisher Scientific, C34554) or Violet (Thermo Fisher Scientific, C34557) Cell Proliferation Kit following manufacturer’s instructions to distinguish them from the sorted T cell populations in the cocultures. The media were changed, and the cells were stained on days 1, 4, 6, 8, 11, 13, 15, 18, 20, and 22.

### Astrocyte-T cell coculture and staining.

The astrocyte cell line CCF-STTG1 (ATCC) was grown in RPMI-1640 medium supplemented with 10% heat-inactivated FBS (Thermo Fisher Scientific, A4766801), 10 mM Hepes buffer solution (Thermo Fisher Scientific, 15630-056), 1 mM sodium pyruvate (Thermo Fisher Scientific, 11360-070), 2 mM L-Glutamine (Thermo Fisher Scientific, 25030-024), and 100 μg/mL penicillin and streptomycin (Thermo Fisher Scientific, 15140-122) at a cell density between 1 × 10^4^ and 2 × 10^5^ cells/cm^2^. Twenty-four hours before the start of the coculture experiments, the astrocytes were plated in a flat bottom 96-well plate (Corning, 10695951) at 10,000 cells per well in 100 μL media. Subsequently, PBMCs were thawed, rested, and stimulated with heat-inactivated HTLV-1 for 12 hours, and CD4^+^ SP, CD8^+^ SP, and DP T cells were sorted as previously described. In total, 5,000 T cells were placed in coculture with the astrocytes for 36 hours. Subsequently, the media and nonadhesive T cells were removed from the wells, the wells were washed with PBS, and 50 μL Accutase (Invitrogen, 00-4555-56) was added for 15 minutes to detach the astrocytes. Astrocytes were stained with annexin V (BioLegend, 640918) following the manufacturer’s specifications. The astrocytes were stained and fixed as previously described. Astrocytes were identified by flow cytometry as CD3^–^ cells.

### Statistics.

Graphs were drawn using R V2022.12.0+353. Data presented in box-and-whisker plots summarize the median of the data; the box extends from the 25th percentile to the 75th percentile (Q1–Q3), and the whiskers connect the remaining lowest and highest 25%. Data were tested for normality using Shapiro-Wilk normality test. Statistical significance for normally distributed data was calculated using a 2-tailed *t* test, and nonparametric data were analyzed using the Wilcoxon signed-rank test. Significance was corrected for multiple comparisons using the Holm method. For data in which we only made predefined comparisons rather than all possible comparisons between groups, we did not correct for multiple comparisons to avoid false negatives and have stated the comparisons made in the figure legends (68).

### Study approval.

All participants provided informed consent before blood draw. Blood samples were obtained through the Communicable Diseases Research Tissue Bank (NRES ID: 20/SC/0226).

### Data availability.

All data presented in this manuscript are available n the Supporting Data Values file. These data include all plotted points and data that were used to calculate any means stated. Patients are referred to by anonymous 3-letter codes.

## Author contributions

AKM performed experiments, analyzed the data, and wrote the manuscript; AA and NG performed experiments and gave advice on the experimental design; YT provided the Tax LT-4 antibody and advised on Tax staining experiments; AA and CRMB provided the primers and probes for ddPCR and advised on ddPCR protocols; GPT provided HTLV-1 AC and HAM patient samples and advised on the clinical aspects of the project; and MDV designed the study, analyzed data, wrote the manuscript, and obtained funding. All authors revised and contributed to the editing of the manuscript.

## Supplementary Material

Supplemental data

Supporting data values

## Version 1 01/09/2024

Electronic publication
