# Supplementary material for: HTLV-1 induces an inflammatory CD4+CD8+ T cell population in HTLV-1–associated myelopathy
Source: JCI Insight. 2024 Jan 9;9(1):e173738. doi: 10.1172/jci.insight.173738 (PMC10906466; doi:10.1172/jci.insight.173738)
Supplement: Supplemental data [file jciinsight-9-173738-s143.pdf]

Supplementary Table 1- Participant Characteristics

Disease Category	UC	AC lPVL	AC hPVL	HAM
**Participant Number**	28	35	24	14
**Age (years)- mean, range**	43.6, 24-68	53.9, 24-79	54.0, 27-81	66.4, 52-88
**Sex – number, percentage**				
**Male**	7, 25%	8, 23%	6, 27%	3, 27%
**Female**	21, 75%	27, 77%	22, 73%	11, 73%
**PVL (% of PBMCs)- mean, range**	NA	0.65, Undetected-2.92	9.65, 3.16-22.89	12.62, 3.51-53.12

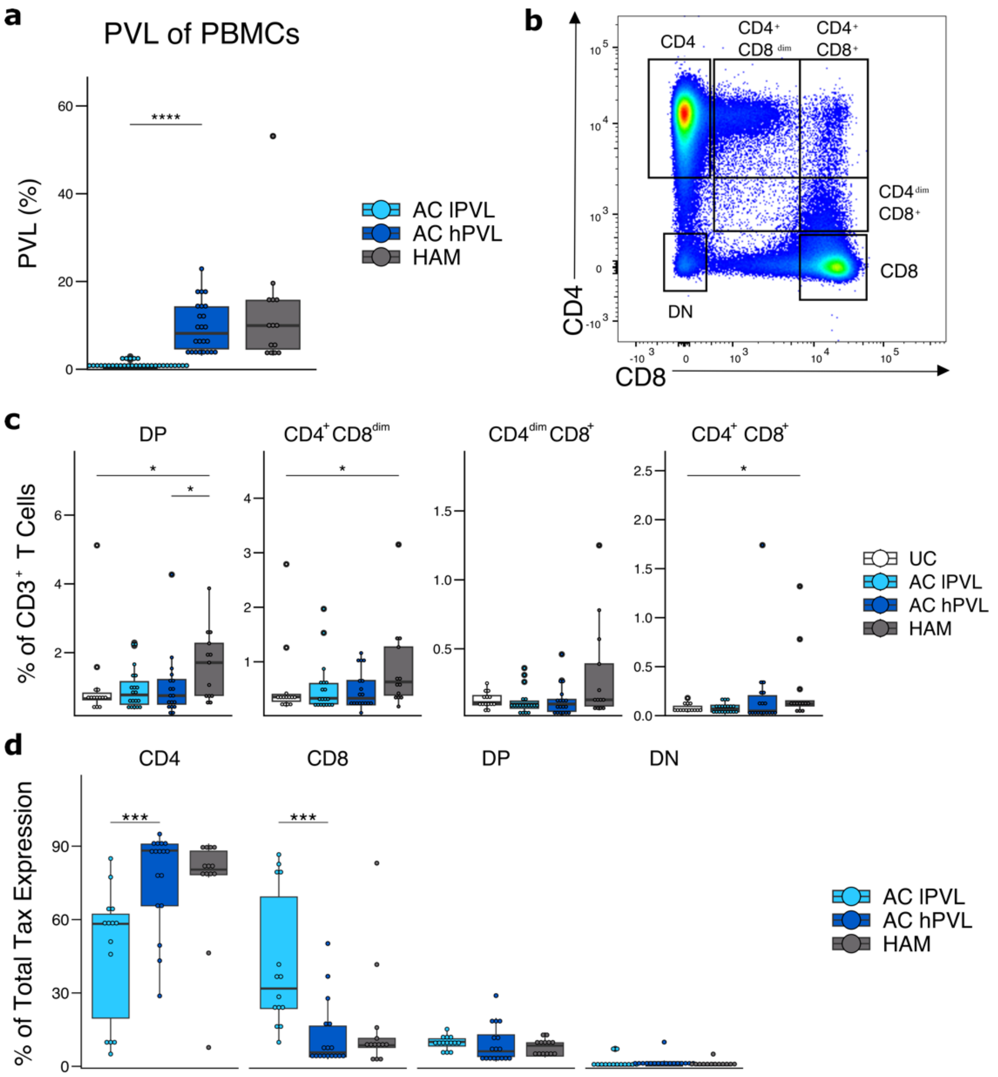

Supplementary Figure 1. PVL of PBMCs and DP T cells gating and infection. a. Box and whisker plot showing the HTLV-1 PVL in total PBMCs (*n*= 20 AC lPVL, *n*=18 AC hPVL, *n*=13 HAM). Representative flow plot (**b**) and box and whisker plot (**c**) showing the frequency of DP T cell sub-populations, $CD4^{+}CD8^{dim}$, $CD4^{+}CD8^{+}$ ($CD4^{bright}CD8^{bright}$), and $CD4^{dim}CD8^{+}$ in different groups (*n*= 14 UC, *n*=19 AC lPVL, *n*=18 AC hPVL, and *n*=13 HAM). **d.** Box and whisker plot showing the proportion of Tax expression contributed by each T cell population in different HTLV-1-infected groups (*n*= 15 AC lPVL, *n*=16 AC hPVL, *n*=13 HAM). **a, c, d)** Wilcoxon signed-rank unpaired test; for **a** and **d** comparisons were made between AC lPVL and AC hPVL, and AC hPVL and HAM. For **c** comparisons were made between UC and AC lPVL, AC lPVL and AC hPVL, UC and HAM, and AC hPVL and HAM; *P<0.05, **P<0.01, ***P<0.001, ****P<0.0001.

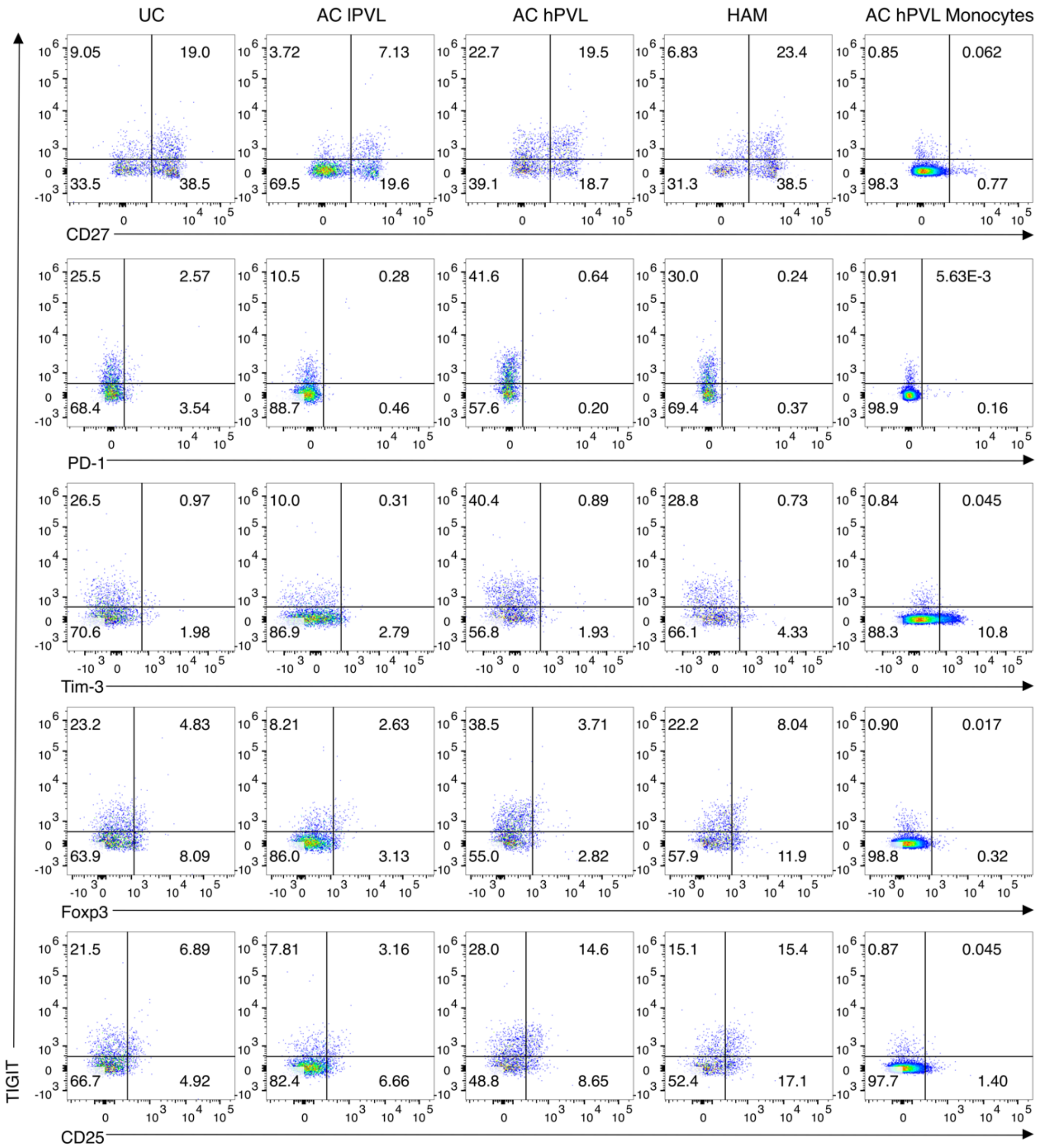

Supplementary Figure 2. ***Ex vivo*** **phenotyping.** Representative flow plots of DP T cells from UCs and HTLV-1-infected groups expressing various *ex vivo* markers.

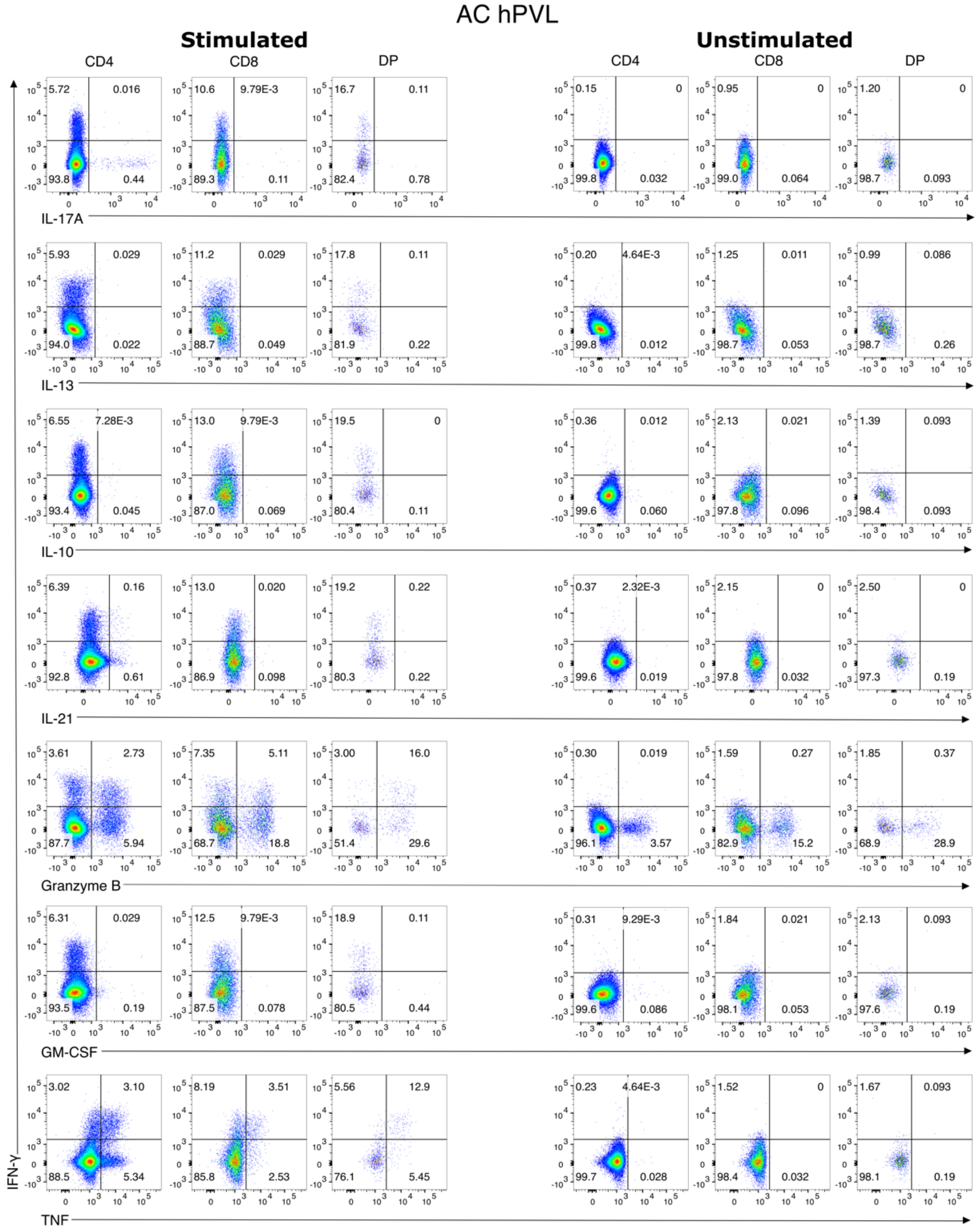

Supplementary Figure 3. Cytokine phenotyping in AC hPVL. Representative flow plots showing cytokine expression by DP T cells from an AC hPVL after stimulation with PMA and ionomycin for 4 hours.

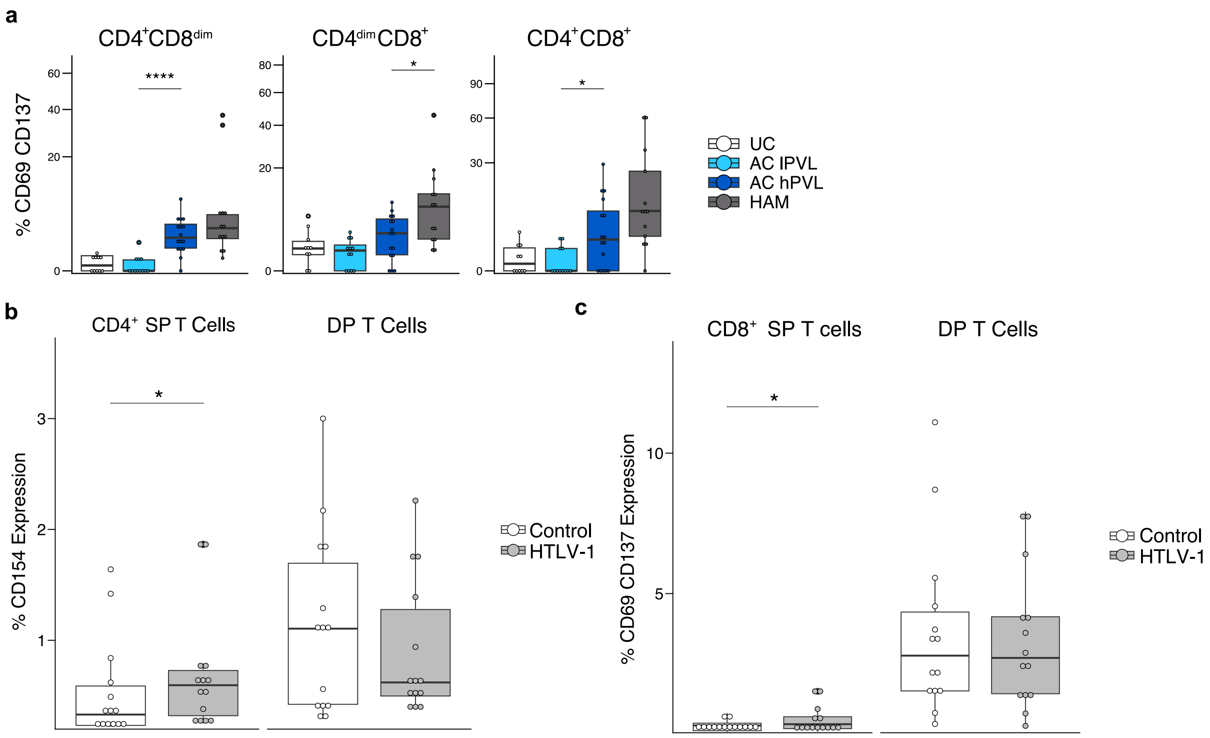

Supplementary Figure 4. Activation marker expression. a. Box and whisker plot comparing the frequency of CD69$^+$CD137$^+$ cells in DP T cells subpopulations between patient groups (*n*= 11 UC, *n*= 12 AC lPVL, *n*=16 AC hPVL, *n*=13 HAM). **b.** Box and whisker plot showing CD154 expression by CD4$^+$ SP and DP T cells after stimulation with heat-inactivated HTLV-1 or a control (*n*=14). **c.** Box and whisker plot showing CD69 and CD137 co-expression by CD8$^+$ SP and DP T cells after stimulation with heat-inactivated HTLV-1 or a control (*n*=14). **a)** Wilcoxon signed-rank unpaired test for comparisons between UC and AC lPVL, AC lPVL and AC hPVL, and AC hPVL and HAM**, b and c)** Wilcoxon signed-rank paired test; *P<0.05, **P<0.01, ***P<0.001, ****P<0.0001.

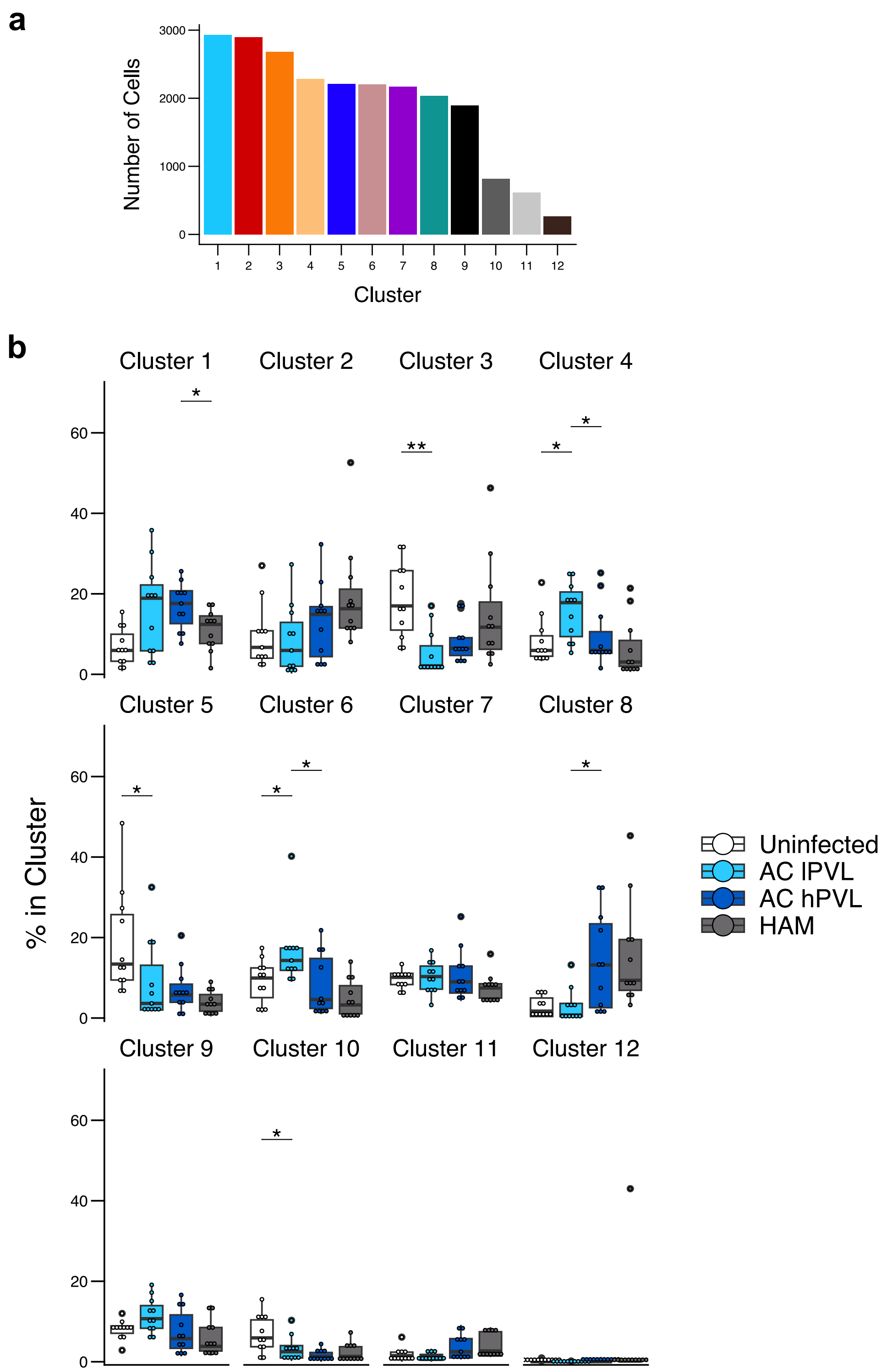

Supplementary Figure 5. Phenograph clusters. a. Bar plot showing the number of DP T cells in each of the 12 clusters identified by Phenograph. **b.** Box and whisker plot showing the percentage of DP T cells from each clinical group in the different Phenograph clusters. Each dot represents an individual (*n*=11 per group). Wilcoxon signed-rank unpaired test, *P<0.05, **P<0.01, ***P<0.001 for comparisons between UC and AC lPVL, AC lPVL and AC hPVL, AC hPVL and HAM.

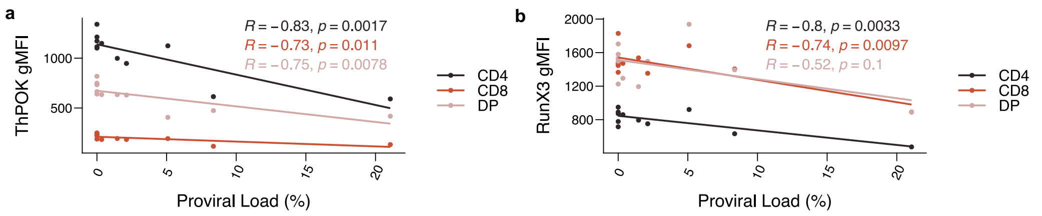

Supplementary Figure 6. ThPOK and RunX3 and PVL correlation. a. Line graph showing the expression of ThPOK with PVL in UCs and ACs (*n*=11). **b.** Line graph showing the expression of RunX3 with PVL in UCs and ACs (*n*=11). **a and b)** Pearson correlation coefficient (R) and *p* value.
